# 2,3-Dihydroferroceno[3,4]pyrrolo[2,1-b]thiazol-5(8b)-ones: Synthesis, Structure and DFT Study on the Mechanism of Chemo- and Diastereoslective Annulations of (*S*_p_)-2-Formylferrocenecarbonyl Fluoride and (*S*_p_)-2-Formylferrocenecarboxylic Acid

**DOI:** 10.3390/molecules26051420

**Published:** 2021-03-05

**Authors:** Zoltán Kovács, Antal Csámpai

**Affiliations:** Department of Organic Chemistry, Eötvös Loránd University (ELTE), Pázmány P. sétány 1/A, H-1117 Budapest, Hungary; khrvtal@gmail.com

**Keywords:** ferrocene, thiazolidine, annulation, lactame, carboxyl-activation, absolute configuration, relative configuration, conformation, diastereoselectivity, reaction mechanism, DFT modelling, MO analysis

## Abstract

By means of the annulation of easy-to-handle yet sufficiently reactive (*S*_p_)-2-formylferrocenecar- bonyl fluoride with the hydrochlorides of cysteamine and the methyl esters of enentiomer cysteines conducted in dichloromethane at room temperature in the presence of pyridine, the first members of 2,3-dihydroferroceno[3,4]pyrrolo[2,1-b]thiazol-5(8*bH*)-ones with the elements of planar- and central chirality were prepared as single enantiomers. An atom economic procedure was also elaborated for the synthesis of these organometallic heterocycles directly exploring (*S*_p_)-2-formylferrocenecarboxylic acid in situ activated by CDI and TFA, sequentially added to the reaction mixture. The relative and consequently, the absolute, configuration of the isolated diastereomers was determined by NMR measurements supported by DFT structural optimization. On the basis of the results of synthetic control experiments and a series of further DFT modelling studies, including energetic and MO analysis of the iminium intermediates, we propose a mechanism for the thiazolidine-forming annulations that proceed via primary *N*-acylation followed by proton-mediated cyclocondensation and subsequent diastereoselective sulfhydryl-attack on the resulting iminium center.

## 1. Introduction

During the last few decades, ferrocene-containing organic compounds have received remarkable attention focused on their chemical stability, highly structure-dependent redox chemistry and stereochemical characteristics associated with planar chirality allowing an array of applications in material sciences, catalysis and in the research of bioactive molecules [1,2,3]. Bioconjugates of ferrocene are also valuable biomaterials of pronounced interest, as this metallocene unit can serve as a biological marker, sensitive probe, chromophore and redox or catalytic active site [4]. Ferrocenyl group was also used as an essential fragment in design and development of antimalarial lead compounds having potential relevance in drug discovery [5,6]. Among the biological effects, antiproliferative activity seems to be the most relevant one disclosed for ferrocene-based organometallics with an exceptional array of structural diversity as demonstrated by a plethora of reviewed examples [7,8,9], prominently including ferrocene-containing analogues of the non-steroidal selective estrogen receptor modulator hydroxytamoxifen [10] that exhibit outstanding cytotoxic and cytostatic effects on hormone-independent MDA-MB-231 breast tumor cells. In this context, our group have synthetized and characterized diverse ferrocene-containing heterocycles with significant antiproliferative activity [11,12,13,14,15,16]. As a continuation of our program expanding the scope of ferrocene-containing heterocycles with potential biological relevance, we envisaged a preliminary study on the synthetic accessibility and structural characteristics of the first members of ferroceno[3,4]pyrrolo[2,1-b]thiazol-5(8bH)-ones. These organometallics, comprising the elements of planar- and central chirality, represent a novel class of ferrocene-fused heterocycles with well-defined stereostructures [12,17,18,19,20,21,22,23,24,25,26]. The diverse molecular architectures of known ferroceno-heterocycles have been assembled by a variety of synthetic methodologies including conventional organic heterocyclisations [12,17,18,19] and protocols based on transition metal-catalysed CH-activation [20,21], alkyne-mediated annulations [22,23], cross-coupling reactions [24], metal-free radical cyclisation [25] as well as iron-incorporation in the late-stage construction of the ferrocene moiety [26].

Besides the fact that the purely organic pyrrolo[2,1-b]thiazolone skeleton can be considered as a ring homologue of the penicillin core, its functionalized derivatives are also present among potent neuroleptic agents [27,28] and antidiabetic drugs [29]. Non-nucleosidic HIV reverse transcriptase inhibitors were also identified among the group of compounds incorporating this bicyclic residue [30,31]. Moreover, it has also been demonstrated that the replacement of an aromatic nucleus in a certain organic compound for a three-dimensional ferrocene unit with a tunable redox character and significant lipophilicity can lead to products possessing enhanced biological activity relative to that displayed by the purely organic parent molecule [5].

## 2. Results and Discussion

### 2.1. Synthesis and Structural Elucidation of Diastereomeric Planar Chiral Ferroceno[3,4]pyrrolo[2,1-b]thiazol-5(8bH)-ones

**Scheme 1:**
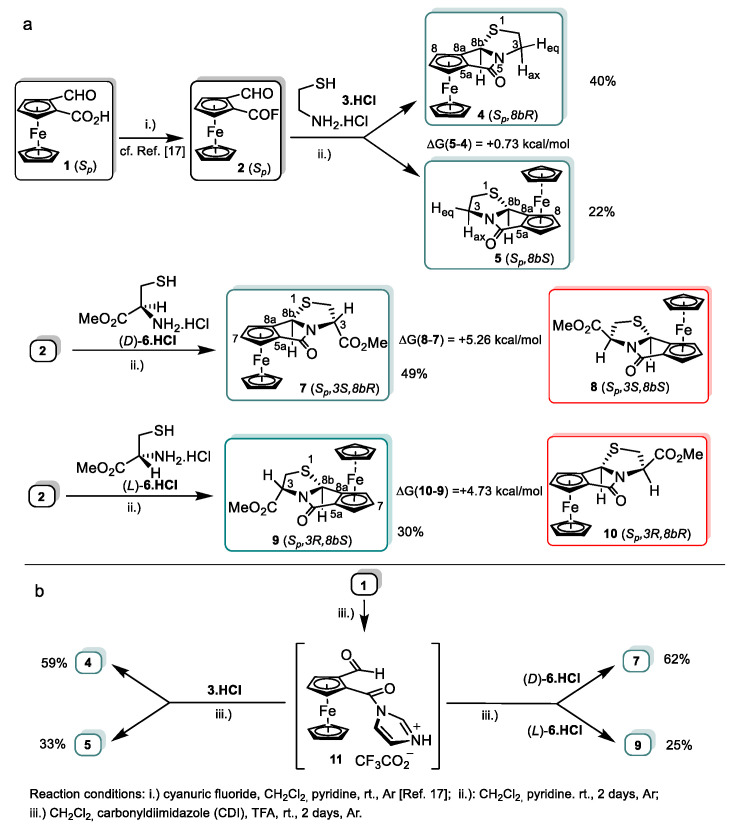
Annulation reactions of cysteamine- and enantiomeric methyl-cysteinate hydrochlorides with (*S*_p_)-2-formylferrocenecarbonyl fluoride (**a**) and (*S*_p_)-2-formylferrocenecarboxylic acid (**b**) characterized by the relative thermodynamics of the potential products obtained by DFT calculations performed at the B3PW91/DGTZVP level of theory. The accessibility of the targeted compounds is highlighted by green and red frames around their structures. The mode of atom numbering presented on compounds **4**, **5**, **7** and **9** is used for the assignment of NMR data.

First, we attempted to perform the annulation reactions of 2-formylferrocene carboxylic acid (Scheme 1) with cysteamine and enantiomeric cysteine methyl esters [**3**, (D)-**6** and (L)-**6**, Scheme 1a] under three documented conditions [31,32,33] successfully applied to the related condensations of 2-formyl- or acylbenzoic acids constructing heterocycles with a 2,3-dihydrothiazolo-[2,3-a]isoindol-5(9bH)-one skeleton; however, these efforts remained in vain, but allowed us to collect the following findings: (i) heating the coupling components under harsh conditions provided by reflux in toluene in the presence of a catalytic amount of toluenesulfonic acid or in xylene, as reported by Mertens et al. [31] and Allin et al. [32], respectively, led to the formation of undefined tarry materials even under argon atmosphere as the probable consequence of a substantially decreased thermal and oxidative stability of the organometallic precursor; (ii) when the mixture of **1** and the hydrochlorid salt of any of the aforementioned mercaptoamine components was stirred in EtOH-water (1:1) at room temperature in the presence of potassium hydrogen carbonate, representing the mild conditions described by Pinho e Melo [33], mixtures of hardly separable interconverting imine- and thiazolidinyl-substituted ferrocene carboxylates were formed as detected by ^1^H-NMR in the evaporated organic (CH_2_Cl_2_) extracts of the reaction mixtures, referring to the resistance of the carboxyl group to participate in lactame-forming cyclisation. Since it has been disclosed in our previous research that (*S*_p_)-2-formylferrocenoylfluoride **2**, readily accessible from **1** (Scheme 1a), can be used as an easy-to-handle yet reactive bis-electrophilic coupling partner in ferroceno[f]pyridazinone-yielding heterocyclisations proceeding via lactame formation [17], we first envisaged testing this reagent as a coupling component in our experiments aimed at the synthesis of the targeted ferroceno[3,4]pyrrolo[2,1-b]thiazol-5(8bH)-ones (Scheme 1a). Thus, coupling reaction **2** with cystemaine hydrochloride (**3.HCl**) conducted for a prolonged time (2 days) at room temperature in dichloromethane in the presence of two equivalents of pyridine serving as base and acyl-transfer catalyst, led to the isolation of an approximately 2:1 mixture of diastereomer products **4** and **5** in acceptable overall yield (62%) as separated by flash column chromatography (**4**: 40%; **5**: 22%). On the other hand, under the same conditions, the annulation reactions of **2** with the hydrochlorides of enantiomeric methyl cysteinates [(D)-**6** and (L)-**6**] afforded lactames **7** and **9** in mediocre and low yields (49% and ca. 30%, respectively). The alternative diastereomers (**8** and **10**) could not be detected by ^1^H-NMR in the crude products contaminated by a mixture of components with undefined structures. The contaminations could be partly, but not completely, removed by flash column chromatography. In this regard, compound **9** proved to be particularly unstable under various conditions of column chromatography using silica as a stationary phase on which a slow decomposition of the desired band was observed. On the other hand, the use of neutral alumina did not allow even a partial separation of the contaminating components. Finally, elution by CH_2_Cl_2_-MeOH (150:1) on silica was found to be a compromise condition affording **9**, with limited purity (ca. 85% by ^1^H-NMR) being the major component of the isolated substance.

In an alternative approach aimed at increasing the yields of the targeted thiazolidines and atom efficiency of the synthetic route, we elaborated an alternative procedure utilizing **1** and the *N*,*S*-dinucleophilic reagents as coupling components in a simple operational step. The activation of the carboxylic group was achieved in DCM at room temperature by 1 equivalent of carbonyldiimidazole (CDI) and a slight excess of TFA (1.1 equiv.) added sequentially to generate protoimidazolinium salt **11** as a reactive intermediate (Scheme 1b). To our delight, thiazolidines **4**, **5** and **9** were accessed in markedly higher yields; however, diastereomer esters **8** and **10** were not obtained even under these conditions and the unstable ester **9** could be isolated in a slightly lower yield with purity (ca. 80–85%) similar to that of the sample obtained by the method using **2** as a dielectrophilic coupling partner.

The ^1^H and ^13^C-NMR data (Supplementary Materials, S2) of the novel ferroceno-fused heterocycles isolated as single diastereomers **4**, **5**, **7** and **9** are consistent with their skeletal structure and relative configuration; however, the following remarks are necessary to make. The presence of the fused ring system is confirmed by the cross peaks originated from three-bond interactions between nucleus pairs H-8b/C-5, H-8b/C-3, H-8b/C-2, H-3_eq,ax_/C-8b (for **4** and **5**) and H-3/C-8b (for **7** and **9**) discernible in the ^1^H-^13^C-HMBC specta. In the ^1^H-NMR spectra of compounds **4** and **5**, due to the anisotropic deshielding effect of the proximal carbonyl group, the signal of the H-3_eq_ proton is significantly downfield shifted relative to that of H-3_ax_ proton, which is involved in NOE interaction with H-8b in both diastereomers. On the other hand, in compound **4** a characteristic NOE was also detected between the η^5^-C_5_H_5_ ligand and H-8b providing an evidence for the endo position of this proton in the organometallic framework and consequently, for the relative configuration of this diastereomer. Besides this type of NOE interaction involving η^5^-C_5_H_5_ ligand and H-8b, in compound **7** an additional NOE was also revealed between H-8b and the OCH_3_ protons referring to the relative configuration of this ester with two stereogenic carbon centers. The considerable ferrocene-anisotropy-induced downfield shifts [34,35] of H-8b signal in the ^1^H-NMR spectra of **4** and **7** relative to those measured for the appropriate diastereomers **5** and **9**, respectively, provide further evidence for “R”-configuration of the C-8b stereogenic center, thus the endo position of the attached H-8b proton in the heterocyclic framework.

### 2.2. Synthetic and DFT Study on the Sequence and Diastereoselectivity of the Thiazolidine-Forming Annulation Reactions

**Scheme 2:**
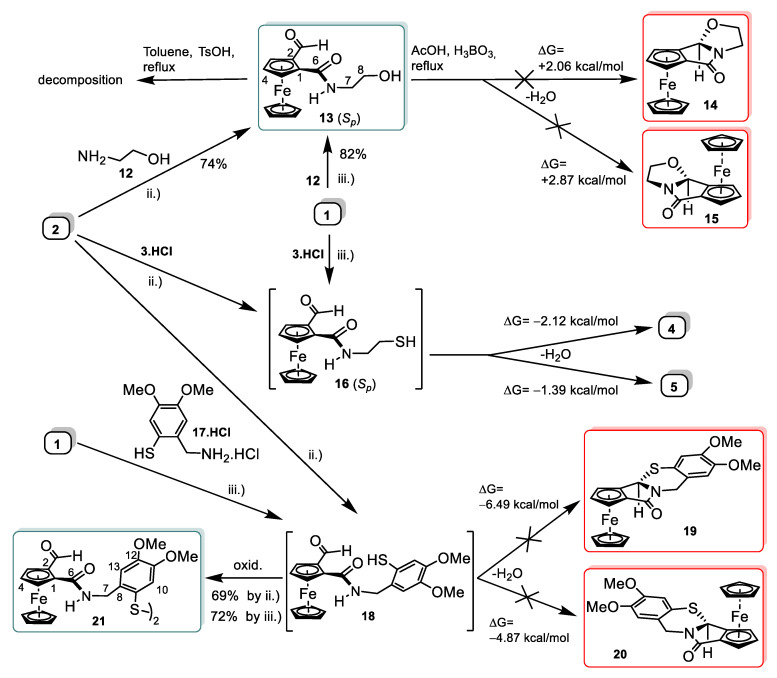
Attempted and realized annulation reactions of selected bifunctional *N*,*O*- and *N*,*S*-donor dinucleophiles with bifunctional electrophilic ferrocene components characterized by the relative thermodynamics of the potential and isolated products obtained by DFT calculations highlighting the significance of overall ring strain and the oxidative susceptibility of a mercaptoamide intermediate. The modes of atom numbering presented on compounds **13** and **21** are used for the assignment of NMR data.

In order to get an insight into the feasibility of the sequential thiazolidine-forming annulation reactions of the highly sensitive organometallic precursors used, we undertook comparative DFT analysis on a selection of possible intermediates assumed to form the basis of the chemoselective transformations with 2-aminoethanol (**12**) and the hydrochloride of 2-(aminomethyl)-4,5-dimethoxybenzenethiol (**17.HCl**), conducted under conditions (ii) and (iii). These reactions afforded amides **13** and **21**, respectively, with an intact formyl group (Scheme 2). Their structures were confirmed by the characteristic ^1^H and ^13^C-NMR signals of the CHO group (10.40 ppm and 196.0 ppm for **13** and 10.38 ppm and 196.1 ppm for **21**) and by the coupling pattern of the NH-CH_2_ fragment displaying triplets of the NH signal and a separated doublet of doublets originated from the diastereotopic protons in the CH_2_ group situated in a planar chiral environment. It is of pronounced importance that **13** proved to be resistant to cyclocondensation to the diastereomers of ferroceno-fused pyrrolo[2,1-b]oxazolones **14** and **15**, even under forced conditions (cf. Scheme 2). Thus, after heating at reflux in acetic acid containing boric acid for 6 h, representing the conditions we have successfully used to promote Biginelli reactions [36] and Pictet-Spengler condensations [37], this amide was recovered a with retained structure in 54% yield from the reaction mixture by means of an aqueous workup. The resistance of **13** to cyclocondensation was rationalized by comparative DFT studies performed by B3PW91 functional [38] employing a DGTZVP basis set [39]. Since our DFT modelling studies were carried out on ferrocene derivatives, we opted for B3PW91 as functional that has been shown to be superior over B3LYP in providing a more reliable and realistic description of bonding parameters in metal-based fragments comprising metal-carbon bond(s) [40]. Thus, in accord with the experimental findings, the theoretical calculations revealed that the hypothetical oxazolidine-forming annulations (**13** → **14** + H_2_O and **13** → **15** + H_2_O) are associated with positive changes in the Gibbs free energy (Scheme 2), while the analogous cyclocondensations of the assumed 2-mercaptoethyl amide intermediate (**16** → **4** + H_2_O and **16** → **5** + H_2_O) were identified as thermodynamically favored transformations (Scheme 2). The spectacular difference in the aptitudes of **13** and **16** to undergo cyclocondensation might be due to the overall ring strain in the fused oxazolidines **14** and **15** being markedly larger than that accumulated in the fused thiazolidines **4** and **5**. On the other hand, the relative thermodynamics calculated for diastereomer pairs **4**/**5**, **7**/**8** and **9**/**10** (Scheme 1a) are also in good agreement with the synthetic results, also indicating a substantially lower degree of selectivity in the generation of **4** and **5**, the products isolated in comparable yields, than in the diastereoselective ester-forming conversions effected by enantiomeric cysteine reagents.

In the 2-mercaptobenzylamine-mediated annulations attempted under conditions (ii) and (iii), the primarily formed amide-based tiophenol intermediate **18** underwent oxidative dimerization to disulfide **21** rather than cyclocondensations to polyfused diastereomeric 1,3-thiazines **19** and **20**, respectively. Besides further confirming the preference of N-acylation over imine formation, this experiment highlighted the significance of possible oxidative transformations that might prevent incorporation of the sulfur atom even in such heterocyclic frameworks of which constructions are highly favored from the thermodynamic point of view (cf. ΔG values calculated for cyclocondensations **18** → **19** + H_2_O and **18** → **20** + H_2_O outlined on Scheme 2). Since the literature data suggest that dichloromethane might have potential to act as an oxidant operating via single electron-transfer (SET) [41], it seems reasonable to assume that oxidative dimerization of **18** was at least partly effected by the solvent used.

**Scheme 3:**
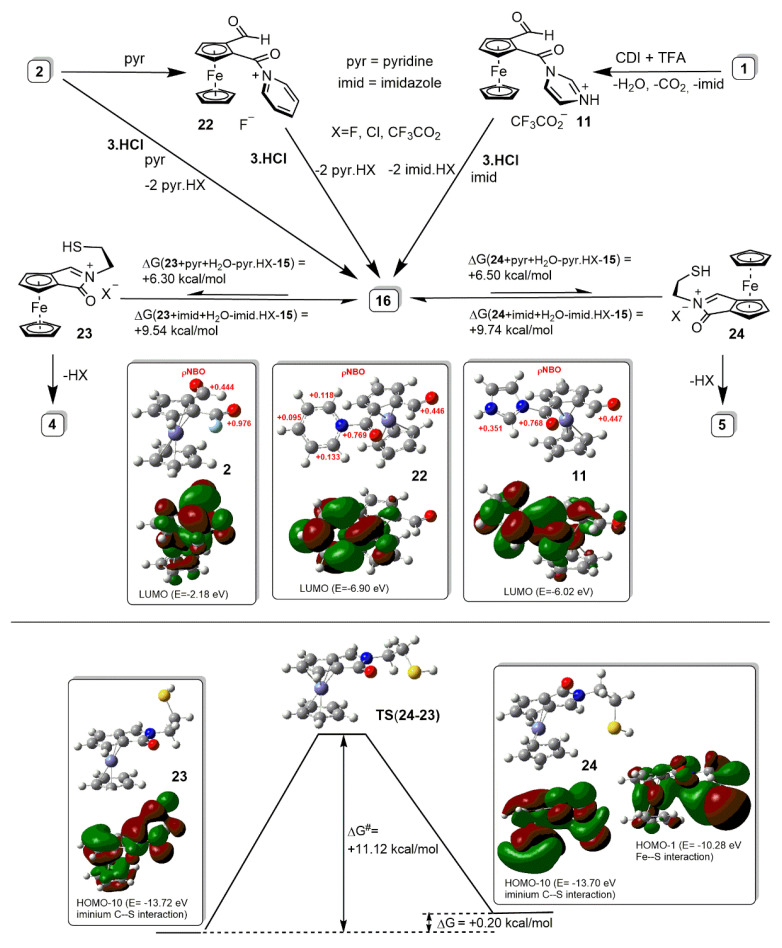
Modelling-based rationalization of the preference of direct as well as pyridine- and CDI-promoted N-acylation and the moderate diastereoselectivity in the cysteamine-mediated cyclisation reactions of dielectrophilic ferrocene precursors attributed to the slight difference in the stability; thus, in the population of the rotamer iminium intermediates rapidly interconverting at room temperature.

Theoretical modelling studies were directed towards rationalization of the crucial chemoselective *N*-acylations, involving **2** as well as the reactive acylium components **22** and **11** generated in situ by pyridine and CDI from **2** and **1**, respectively (Scheme 3). DFT calculations were also used to reveal such plausible pathways of subsequent annulations that can account for the reagent-dependent diastereoselectivity manifested in the experimental product distributions. In order to estimate the relative reactivity of the alternative electrophilic sites in **2**, **22** and **11**, the LUMO delocalization and the local NBO charges [42] were disclosed in their optimized structures as presented on Scheme 3. In acylfluoride **2**, the approximately even LUMO distribution would refer to a highly similar formyl- and fluorocarbonyl reactivities in a purely orbital-controlled elementary step with a donor component, while the calculated NBO charges unambiguously identify the fluorocarbonyl group as the preferred electrophilic site in a nucleophilic attack simultaneously governed by orbital overlaps and Coulombic interactions. In cations **22** and **11** the concentration of LUMO on the *N*-acylpyridinium residue without or a minimal delocalization towards the formyl group along with the results of NBO analysis (Scheme 3) indicate that—in agreement with the experimental findings and the general expectations—the carbonyl group in the cationic fragment is the most reactive site involved in acylpyridinium-mediated acyl-transfer progressing under simultaneous controls of orbital overlap and electrostatic interactions. On the effect of weakly acidic protopyridinium and protoimidazolium salts, the resulting amide type intermediate **16** is assumed to undergo equilibrium cyclocondensation generating reactive diastereomer iminium cations **23** and **24** in low concentrations of similar thermodynamic stability. It must be pointed out here that besides the weakly acidic protoimidazolium salts with lowered efficiency in promoting cation-generation (compare ΔG values calculated for pyr.HX- and imid.HX-mediated cyclisations of **16**: Scheme 3) the slight excess (0.1 equiv.) of TFA present under condition (iii) possibly act as an additional promoter in these crucial conversions. The resulting cationic intermediates **23** and **24** feature intramolecular interactions of the pending sulfhydryl group with the iminium- and ferrocene iron centers as visualized by the appropriate delocalized orbitals presented on Scheme 3. Since the thiazolidine forming cyclisations are certainly irreversible, the moderate but highly similar levels of diastereoselectivity established for the cysteamine-mediated annulations of both the investigated ferrocene precursors (cf. Scheme 1a,b) suggest that, regardless of the reaction conditions used, these transformations proceed via amide **16** as outlined on Scheme 3. Consequently, the comparable yields of **4** and **5** must be correlated with the small difference in the thermodynamic stability of diastereomer rotamers **23** and **24** (Scheme 3) capable of rapidly interconverting via a relatively low energy barrier (11.12 kcal/mol), allowing high rotation frequency of ca. 5 × 10^5^ 1/s at room temperature, as calculated by Eyring equation [43]. The transition state was localized by the QST2 method [44] as a saddle point on the potential energy surface (PES). It can also be assumed that due to the Fe---S interaction disclosed by HOMO-1, the minor iminium component **24** has a somewhat lower tendency to undergo thiazolidine-forming cyclisation as compared to its rotamer counterpart **23**. It must be pointed out here that the ΔG values calculated for iminium-forming cyclodondensations would be substantially decreased by using the highly demanding exact solvent model that takes a number of arbitrarily selected intermolecular interactions into account, contributing to the stability of the resulting iminium cations.

**Scheme 4:**
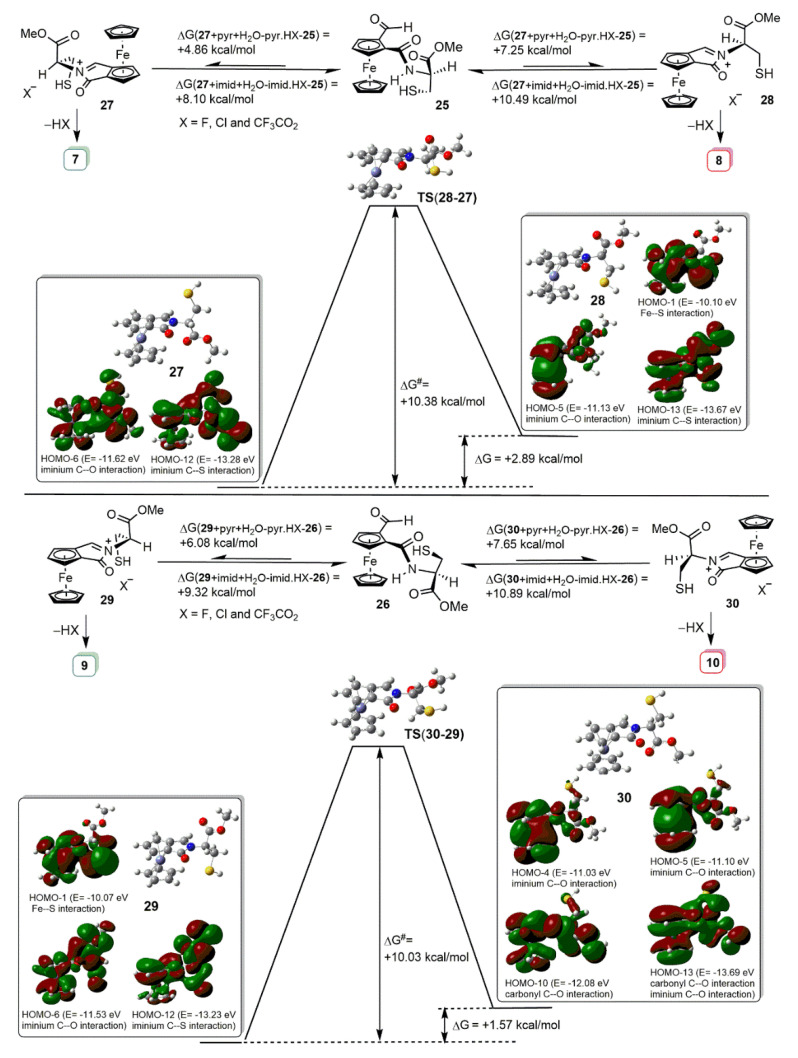
Modelling-based rationalization of the marked diastereoselectivity in the cyclisation reactions mediated by the enantiomeric cysteine reagents attributed to increased differences in the stability, thus, in the population of rotameric pairs of iminium intermediates rapidly interconverting at room temperature.

In order to reveal the reasons for the pronounced diastereoselectivity observed for the annulations effected by enantiomeric cystein reagents, we applied the aforementioned DFT methods to track the reaction pathways closely related to those proposed for the cysteamine-mediated reactions. Accordingly, disclosing thermodynamics of the protopyridinium- and protoimidazolinium-promoted competing equilibrium cyclocondensations of diastereomer amides **25** and **26**, respectively, and all the other components involved were subjected to geometry-optimization and subsequent frequency calculations. We also performed MO analysis on the rotameric pairs of the resulting iminium ions (**27**/**28** and **29**/**30**: Scheme 4). It must be pointed out that, in line with the experimental findings, the population-determining differences in their free energy values were found to be significantly larger than those calculated for iminium rotamers **23** and **24**, the intermediates of thiazolidine-forming annulations progressing with markedly decreased diastereoselectivity. The relative stability, thus, the population distribution of rotameric iminium ion pairs **27**/**28** and **29**/**30** can be markedly influenced by the intramolecular interatomic interactions visualized by the relevant delocalized MO’s of different energy levels as presented on Scheme 3. In these cations, besides the sulfhydryl group, the carbonyl oxygen of the ester group situated on the opposite face of the fused pyrrolidinium plane is also involved in donor–acceptor interactions with the iminium center. However, in **30** featuring extended C---O interactions (cf. HOMO-4, HOMO-5, HOMO-10 and HOMO-13), no delocalized MO could be identified with clearly discernible C---S contact, probably further decreasing the feasibility of the ring closure and terminating the multistep annulation that would finally lead to fused thiazolidine **10**. Finally, it is of note that—in accord with the expectations based on the general view on stereoelectronic interactions—no bonding orbitals delocalized between the aforementioned donor and acceptor sites could unambiguously be identified in the transition state structures presented in Scheme 3 and Scheme 4.

## 3. Materials and Methods

All fine chemicals were obtained from commercially available sources (Merck, Budapest, Hungary), Fluorochem (Headfield, UK), Molar Chemicals (Budapest, Hungary), (VWR, Budapest, Hungary) and were used without further purification. Dichloromethane (DCM) was distilled from then stored on CaH_2_. Merck Kieselgel (230–400 mesh, 60 Å) was used for flash column chromatography. (*S*_p_)-2-Formylferrocenoylfluoride (**2**) was synthesized from 2-formylferrocene carboxylic acid (**1**) using the procedure described in Ref. [17]. Precursor **1** was obtained in four synthetic steps from formylferrocene as reported also in Ref. [17]. Melting points (uncorrected) were determined with a M-560 instrument (Büchi, Essen, Germany). The ^1^H- and ^13^C-NMR spectra were recorded in DMSO-d_6_solution in 5 mm tubes at room temperature (RT), on a Bruker DRX-500 spectrometer (Bruker Biospin, Karlsruhe, Baden Württemberg, Germany) at 500 (^1^H) and 125 (^13^C) MHz, with the deuterium signal of the solvent as the lock and TMS as internal standard (^1^H, ^13^C). The 2D-NOESY, HSQC, and HMBC spectra were obtained by using the standard Bruker pulse programs noesygpphpp (2D phase sensitiveNOESY with gradient pulses in mixing time and purge pulses before relaxation delay d1 for NOESY), hsqcetgp (2D phase sensitive HSQC using Echo/Antiecho-TPPI gradient selection with decoupling during acquisition and using trim pulses in inept transfer) for HSQC and hmbcgpndqf (2D H-1/XHMBC optimized on long range couplings, no decoupling during acquisition using gradient pulses for selection for HMBC). All calculations were carried out using the Gaussian 09 software (Gaussian Incorporation, Pittsburgh, PA, USA) package [45]. The optimized structures are available from the authors.

### 3.1. General Procedure for the Conversions of (S_p_)-2-Formylferrocenoylfluoride **2** with N,S- and N,O-Donor Bifunctional Nucleophilic Reagents **(3.HCl**, (D)**-6.HCl**, (L)**-6.HCl**, **12** and **17.HCl**) by Method (ii)

Under an argon atmosphere, (*S*_p_)-2-formylferrocenoylfluoride (0.520 g, 2 mmol), pyridine (330 μL, 4 mmol) and the bifunctional nucleophilic reagent (2 mmol) were dissolved in DCM (10 mL) stored on CaH_2_ and were previously bubbled through with argon and cooled down to 0 °C (When using 2-aminoethanol as coupling component, 165 μL (2 mmol) of pyridine was added to DCM). The resulting mixture was allowed to warm up to room temperature and was stirred for 2 days under argon. After completion of the reaction, the mixture was quenched with water (40 mL) and extracted with DCM (5 × 25 mL). The combined organic phase was washed with brine, dried on Na_2_SO_4_, and evaporated. The residue was purified by flash chromatography on silica using DCM-MeOH mixtures (DCM and MeOH ratio was varied between 100:1 and 150:1). The collected yellow-to-orange bands were checked by TLC and evaporated to dryness. The oily residue was solidified with methanol-water, filtered off and dried under argon.

#### 3.1.1. (*S*_p_,8bR)-2,3-Dihydroferroceno[3,4]pyrrolo[2,1-b]thiazol-5(8bH)-one (**4**)

Yellow solid. Yield: 0.238 g (40%). R_f_ = 0.55 (DCM-MeOH 50:1). M.p. 104–107 °C. ^1^H-NMR (DMSO-d_6_): 6.00 (s, 1H, H-8b); 4.84 (dd, J = 2.5 Hz and 1.0 Hz, 1H, H-6); 4.59 (br d, J = 2.5 Hz, 1H, H-8); 4.39 (t, J = 2.5 Hz, 1H, H-7); 4.27 (m, 1H, H-3_eq_); 4.25 (s, 5H, η^5^-C_5_H_5_); 3.48 and 3.22 (2 x m, 2 x 1H, H-2); 3.12 (m, 1H, H-3_ax_). ^13^C-NMR (DMSO-d_6_): 175.5 (C-5); 96.1 (C-8a); 75.3 (C-5a); 73.9 (C-7); 71.5 (η^5^-C_5_H_5_); 65.8 (C-6); 65.2 (C-8b); 62.6 (C-8); 45.6 (C-3); 38.2 (C-2). Anal. calcd. for C_14_H_13_FeNOS: C, 56,21%; H, 4.38%; N, 4.68%; S, 10.72%. Found: C, 56,37%; H, 4.20%; N, 4.76%; S, 10.80%.

#### 3.1.2. (*S*_p_,8bS)-2,3-Dihydroferroceno[3,4]pyrrolo[2,1-b]thiazol-5(8bH)-one (**5**)

Yellow solid. Yield: 0.130 g (22%). R_f_ = 0.50 (DCM-MeOH 50:1). M.p. 119–122 °C. ^1^H-NMR (DMSO-d_6_): 5.58 (s, 1H, H-8b); 4.60 (dd, J=2.4 Hz and 1.0 Hz, 1H, H-8); 4.58 (dd, J = 2.4 Hz and 1.0 Hz, 1H, H-6); 4.43 (t, J = 2.5 Hz, 1H, H-7); 4.34 (s, 5H, η^5^-C_5_H_5_) 4.10 (dt, J = 11.4 Hz and 5.6 Hz, 1H, H-3_eq_); 3.26 (~t, J~6 Hz, 2H, H-2); 3.07 (dt, J = 11.4 Hz and 5.6 Hz, 1H, H-3_ax_); ^13^C-NMR (DMSO-d_6_): 172.7 (C-5); 97.8 (C-8a); 81.5 (C-5a); 73.5 (C-7); 70.8 (η^5^-C_5_H_5_); 64.9 (C-8b); 64.4 (C-6); 61.9 (C-8); 45.8 (C-3); 35.4 (C-2). Anal. calcd. for C_14_H_13_FeNOS: C, 56,21%; H, 4.38%; N, 4.68%; S, 10.72%. Found: C, 56,43%; H, 4.53%; N, 4.57%; S, 10.64%.

#### 3.1.3. Methyl (*S*_p_,3S, 8bR)-5-oxo-2,3,5,8b-tetrahydroferroceno[3,4]pyrrolo[2,1-b]thiazol-3-carboxylate (**7**)

Yellow solid. Yield: 0.348 g (49%). R_f_ = 0.45 (DCM-MeOH 50:1). M.p. 98–101 °C. ^1^H-NMR (DMSO-d_6_): 6.12 (s, 1H, H-8b); 4.97 (dd, J = 8.1 Hz and 5.0 Hz, 1H, H-3); 4.94 (dd, J = 2.5 Hz and 1.0 Hz, 1H, H-6); 4.71 (dd, J = 2.5 Hz and 1.0 Hz, 1H, H-8); 4.46 (t, J = 2.5 Hz, 1H, H-7); 4.29 (s, 5H, η^5^-C_5_H_5_); 3.78 (s, 3H, CO_2_CH_3_); 3.69 (dd, J = 11.3 Hz and 8.1 Hz, 1H, H-2_b_); 3.54 (dd, J = 11.3 Hz and 5.1 Hz, 1H, H -2_a_). ^13^C-NMR (DMSO-d_6_): 174.4 (C-5); 171.5 (CO_2_CH_3_); 94.5 (C-8a); 74.4 (C-7); 74.3 (C-5a); 71.9 (η^5^-C_5_H_5_); 66.7 (C-6); 65.8 (C-8b); 63.2 (C-8); 58.0 (C-3); 53.5 (CO_2_CH_3_); 39.5 (C-2). Anal. calcd. for C_16_H_15_FeNO_3_S: C, 53.80%; H, 4.23%; N, 3.92%; S, 8.92%. Found: C. 53,95%; H, 4.00%; N, 4.03%; S, 8.86%.

#### 3.1.4. Methyl (*S*_p_,3R, 8bS)-5-oxo-2,3,5,8b-tetrahydroferroceno[3,4]pyrrolo[2,1-b]thiazol-3-carboxylate (**9**)

Yellow solid. Yield: 0.250 g (ca. 30% as calculated for the content of **9** with the assumption that the contaminating components have similar molecular weights). R_f_ = 0.55 (DCM-MeOH 50:1). M.p. 87–94 °C. ^1^H-NMR (DMSO-d_6_): 5.67 (s, 1H, H-8b); 5.05 (dd, J = 7.1 Hz and 4.2 Hz, 1H, H-3); 4.67 (br d, J = 2.5 Hz, 1H, H-6); 4.62 (br d, J = 2.5 Hz 1H, H-8); 4.49 (t, J = 2.5 Hz, 1H, H-7); 4.36 (s, 5H, η^5^-C_5_H_5_); 3.69 (s, 3H, CO_2_CH_3_); 3.62 (dd, J = 11.4 Hz and 7.1 Hz, 1H, H-2_b_); 3.48 (dd, J = 11.4 Hz and 4.2 Hz, 1H, H-2_a_). ^13^C-NMR (DMSO-d_6_): 172.0 (C-5); 171.0 (CO_2_CH_3_); 97.1 (C-8a); 80.4 (C-5a); 74.0 (C-7); 71.0 (η^5^-C_5_H_5_); 65.4 (C-8b); 64.2 (C-8); 62.3 (C-6); 58.5 (C-3); 53.5 (CO_2_CH_3_); 38.4 (C-2).

#### 3.1.5. (*S*_p_)-2-Formyl-N-(2-hydroxyethyl)ferrocene-carboxamide (**13**)

Yellow solid. Yield: 0.445 g (74%). R_f_ = 0.33 (DCM-MeOH 50:1). M.p. 139–142 °C. ^1^H-NMR (DMSO-d_6_): 10.40 (s, 1H, CHO); 8.45 (t, J = 5.5 Hz, 1H, NH); 5.30 (br s, 1H, H-5); 4.94 (br s, 1H, H-3); 4.83 (br s, 1H, H-4); 4.71 (t, J = 6.0 Hz, 1H, OH); 4.31 (s, 5H, η^5^-C_5_H_5_); 3.50 (~qa, J~6 Hz, 2H, H-8). ^13^C-NMR (DMSO-d_6_): 196.0 (CHO); 169.0 (C-6); 79.9 (C-2); 79.2 (C-1); 75.1 (C-5); 74.2 (C-4); 73.1 (C-3); 72.1 (η^5^-C_5_H_5_); 60.8 (C-8); 42.7 (C-7). Anal. calcd. for C_14_H_15_FeNO_3_: C, 55.84%; H, 5.02%; N, 4.65%. Found: C, 55.75%; H, 4.94%; N, 4.80%.

#### 3.1.6. (*S*_p_,*S*_p_)-*N*,*N*′-((Disulfanediylbis(4,5-dimethoxy-2,1-phenylene))bis(methylene))bis(2-formylferro-cene-carboxamide) (**21**)

Yellow solid. Yield: 0.603 g (69%). R_f_ = 0.25 (DCM-MeOH 50:1). M.p. 163–165 °C. ^1^H-NMR (DMSO-d_6_): 10.38 (s, 1H, CHO); 8.90 (t, J = 5.7 Hz, 1H, NH); 7.11 (s, 1H, H-13); 7.00 (s, 1H, H-10); 5.34 (dd, J = 2.5 Hz and 1.0 Hz, 1H, H-5); 5.00 (dd, J = 2.5 Hz and 1.0 Hz, 1H, H-2); 4.88 (t, J = 2.5 Hz, 1H, H-4); 4.53 (dd, J = 14.9 Hz and 5.7 Hz, 1H, H-7_A_); 4.45 (dd, J = 14.9Hz and 5.7 Hz, 1H, H-7_B_); 4.32 (s, 5H, η^5^-C_5_H_5_); 3.80 (s, 3H, 12-OCH_3_); 3.69 (s, 3H, 11-OCH_3_). ^13^C-NMR (DMSO-d_6_): 196.1 (CHO); 168.8 (C-6); 150.8 (C-11); 148.8 (C-12); 135.1 (C-8); 125.9 (C-9); 117.9 (C-10); 113.4 (C-13); 79.5 (C-2); 79.1 (C-1); 76.0 (C-5); 74.4 (C-4); 73.7 (C-3); 72.0 (η^5^-C_5_H_5_); 56.6 and 56.5 (11-OCH_3_ and 12-OCH_3_); 41.7 (C-7). Anal. calcd. for C_42_H_40_Fe_2_N_2_O_8_S_2_: C, 57.55%; H, 4.60%; N, 3.20%; S, 7.31%. Found: C, 57.49%; H, 4.68%; N, 3.27%; S, 7.35%.

### 3.2. General Procedure for the Conversions of (S_p_)-2-Formylferrocenecarboxylic acid **1** with N,S- and N,O-Donor Bifunctional Nucleophilic Reagents (**3.HCl**, (D)-**6.HCl**, (L)-**6.HCl**, **12** and **17.HCl**) by Method (iii)

Under an argon atmosphere, (*S*_p_)-2-formylferrocenecarboxylic acid (0.516 g, 2 mmol) and CDI (0.325 g, 2 mmol) were dissolved in DCM (10 mL) stored on CaH_2_ and previously bubbled through with argon. After completion of acylimidazolide formation (accompanied by CO_2_ evolution), first TFA (170 μL 2.2 mmol), then immediately the bifunctional nucleophilic reagent (2 mmol), were sequentially added to the solution. The resulting mixture was stirred at room temperature for 2 days under argon then quenched with aqueous 5% sodium bicarbonate solution (40 mL) and extracted with DCM (5 × 25 mL). The combined organic phase was washed with brine, dried on Na_2_SO_4_, and evaporated. The residue was purified by column chromatography and subsequent crystallization as described in **3.1.** to obtain compounds **4** (0.351 g, 59%), **5** (0.196 g, 33%), **7** (0.440 g, 49%), **9** (0.205 g, ca. 25% as calculated for the content of **9** with the assumption that the contaminating components have similar molecular weights), **13** (0.493 g, 82%) and **21** (0.630 g, 72%). The analytical and spectral data of these products—within the experimental error—were identical to those measured for the samples isolated after the reactions conducted under the conditions of method (ii).

## 4. Conclusions

In the course of a preliminary synthetic study, two simple procedures were applied for the construction of the first representatives of novel stereoisomer ferroceno-fused pyrrolo[2,1-b]thiazolones of potential interest in a fragment-based design aimed at the identification of such molecular scaffolds that can be explored in the development of novel bioactive agents. The mechanisms of the multistep annulations proposed on the basis of DFT modelling studies and control experiments, accounting for the primary amide-formation and diastereoselectivity, were explored in the design and optimization of further, more efficient procedures, providing easier access to the targeted compounds, presented in this contribution, and to related heterocyclic organometallics with potential interest in pharmaceutical research and other applications.

## Data Availability

The data presented in this study are available in this article and in the Supplementary Materials.

## Supplementary Materials

The following are available online. Details of theoretical calculations (**S1**) Copies of ^1^H- and ^13^C-NMR spectra (**S2**): ^1^H-NMR of **4**; ^13^C-NMR of **4;** HSQC of **4**; HMBC of **4**: NOESY of **4**; ^1^H-NMR of **5**; ^13^C-NMR of **5;** HMBC of **5;** ^1^H-NMR of **7**; ^13^C NMR of **7;** HSQC of **7**; HMBC of **7**: NOESY of **7;** ^1^H-NMR of **9**; ^13^C-NMR of **9;** HMBC of **9**; ^1^H-NMR of **13**; ^13^C-NMR of **13**; ^1^H-NMR of **21**; ^13^C-NMR of **21**; HMBC of **21** XYZ coordinates of the following optimized structures obtained by DFT modelling **(S3**): **2**; **4**; **5**, 7-**11**; **13**-**16**; **18**-**20**; **22**-**30**; **TS(24-23)**; **TS(28-27)**; **TS(30-29)**.

## Sample Availability

Samples of the compounds reported in this contribution are available from the authors.
