# Supplementary material for: 2,3-Dihydroferroceno[3,4]pyrrolo[2,1-b]thiazol-5(8b)-ones: Synthesis, Structure and DFT Study on the Mechanism of Chemo- and Diastereoslective Annulations of (Sp)-2-Formylferrocenecarbonyl Fluoride and (Sp)-2-Formylferrocenecarboxylic Acid
Source: Molecules. 2021 Mar 5;26(5):1420. doi: 10.3390/molecules26051420 (PMC7961964; doi:10.3390/molecules26051420)
Supplement: Supplementary file 1 [file molecules-26-01420-s001.pdf]

2,3-Dihydroferroceno[3,4]pyrrolo[2,1-b]thiazol-5(8b)-ones: synthesis, structure and DFT study on the mechanism of chemo- and diastereoslective annulations of (S_p)-2-formylferrocenecarbonyl fluoride and (S_p)-2-formylferrocenecarboxylic acid

Zoltán Kovács [1] and Antal Csámpai [1*]

[2] Department of Organic Chemistry, Eötvös Loránd University (ELTE) Pázmány P. sétány 1/A, H-1117 Budapest, Hungary; khrvtal@gmail.com (Z.K.)

* Correspondence: csampai@caesar.elte.hu; Tel.: +36-01-372-2500 (ext. 6591)

Supplementary Material

Content:

S.1. Details of theoretical calculations. pp. 1 – 1

S.2. Copies of the NMR spectra of the products pp. 2 – 13

S.3. XYZ coordinates of the optimized structures pp. 14 – 37

S.1.

In the course of DFT calculations the optimised structures were localised as local minima on the potential energy surface (PES) by B3PW91 functional employing DGTZVP basis set. On the optimised structures frequency calculations were carried out employing the same functional and basis to obtain the Gibbs free energy values. Using QST2 method transition states were localised as saddle points connecting two local minima on the PES. The vibrational spectrum calculated for these structures comprise one imaginary frequency providing evidence for their TS character associated with saddle point position on the PES. In the course of the calculations molecular orbitals (MO's) were accumulated in the checkpoint files. Using downloaded „formchk.exe" utility the checpoint files were then converted into a formated file suitable for visualisation. From the full set of MO's the ones displaying the appropriate interatomic contacts were selected and visualised. Natural bond analysis was run simultaneously with geometry optimisation providing NBO charges. All calculations were performed by the Gaussian 09 software (Gaussian Incorporation, Pittsburgh, U.S.) package.

S.2.

^{1}H NMR of **4**

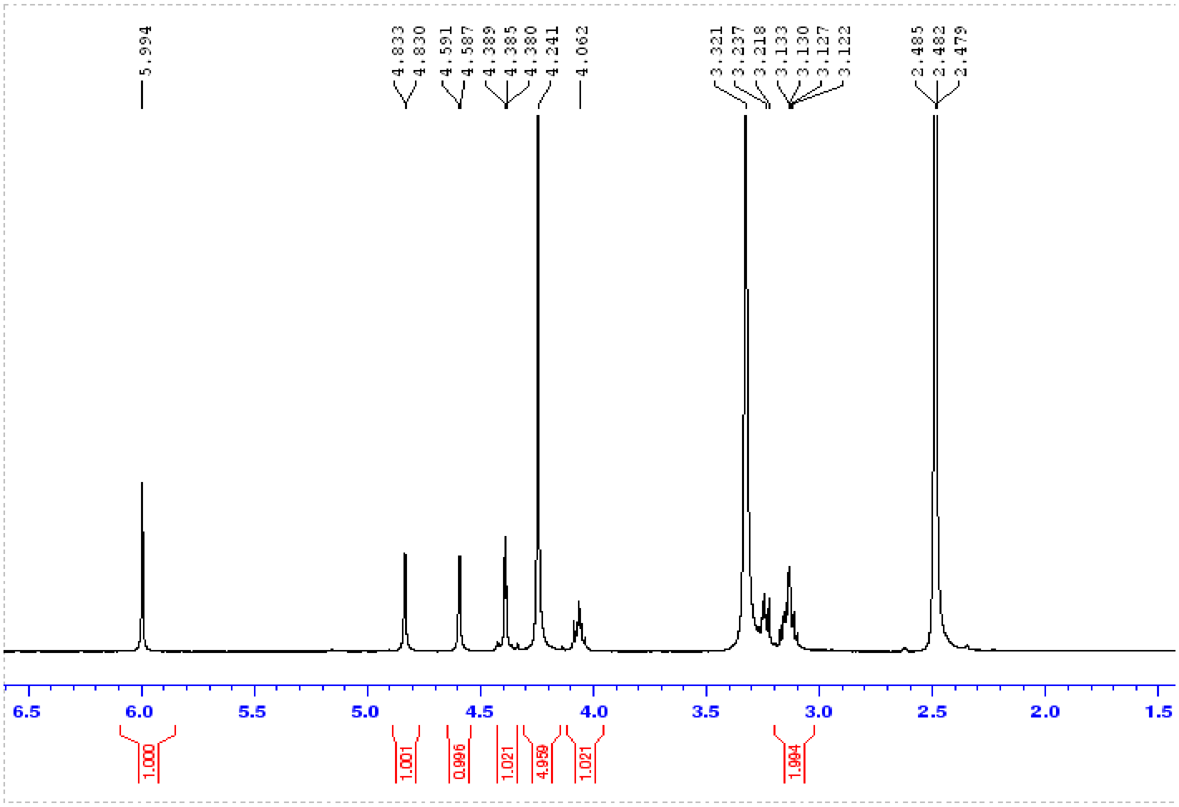

^{13}C NMR of **4**

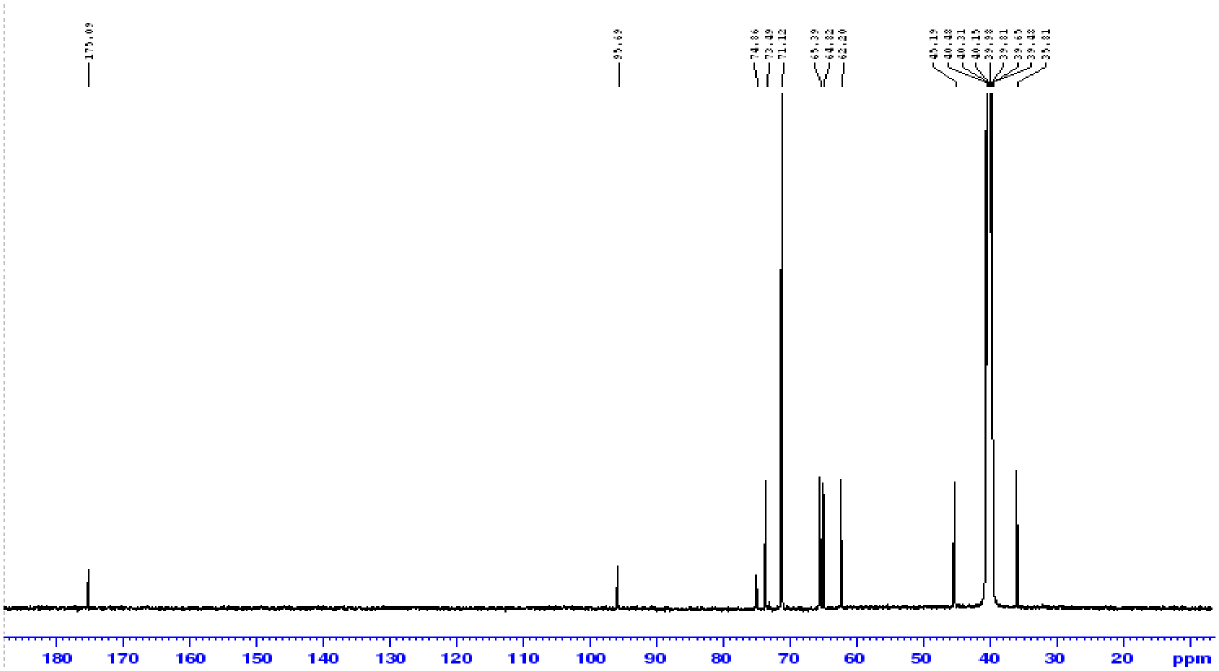

HSQC of **4**

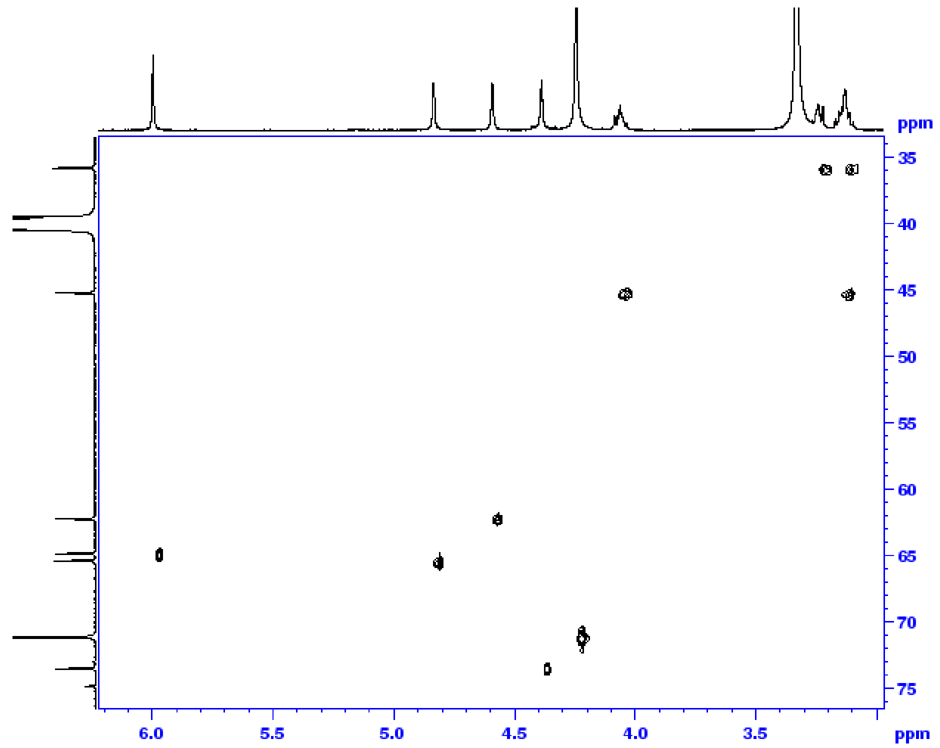

HMBC of **4**

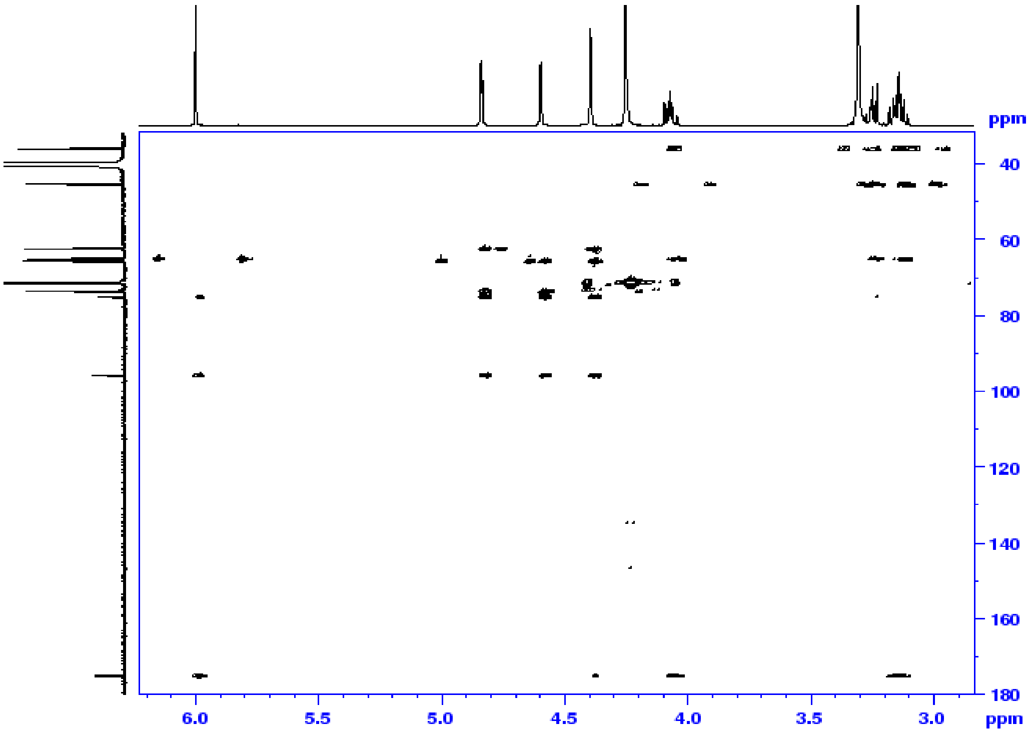

NOESY of **4**

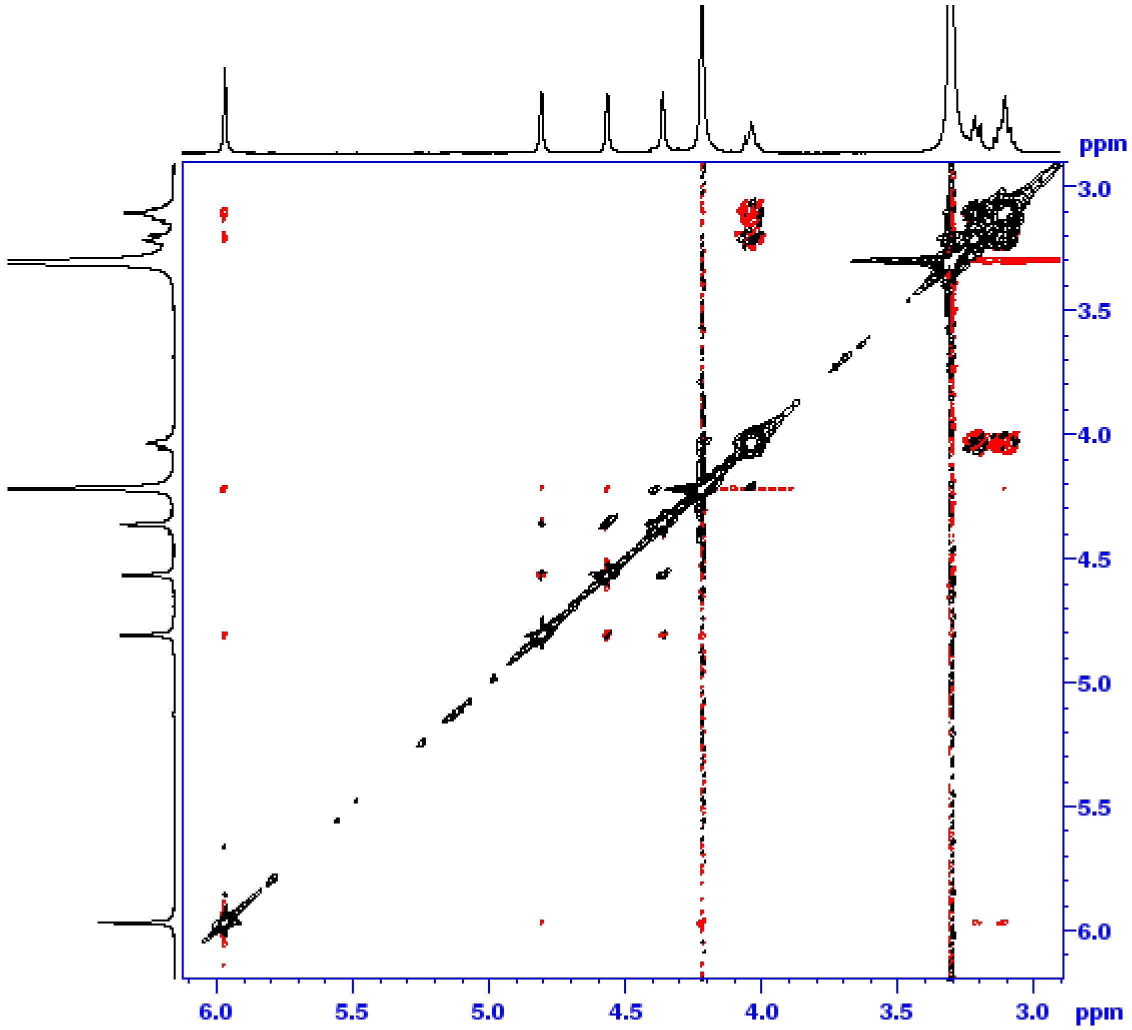

^{1}H NMR of **5**

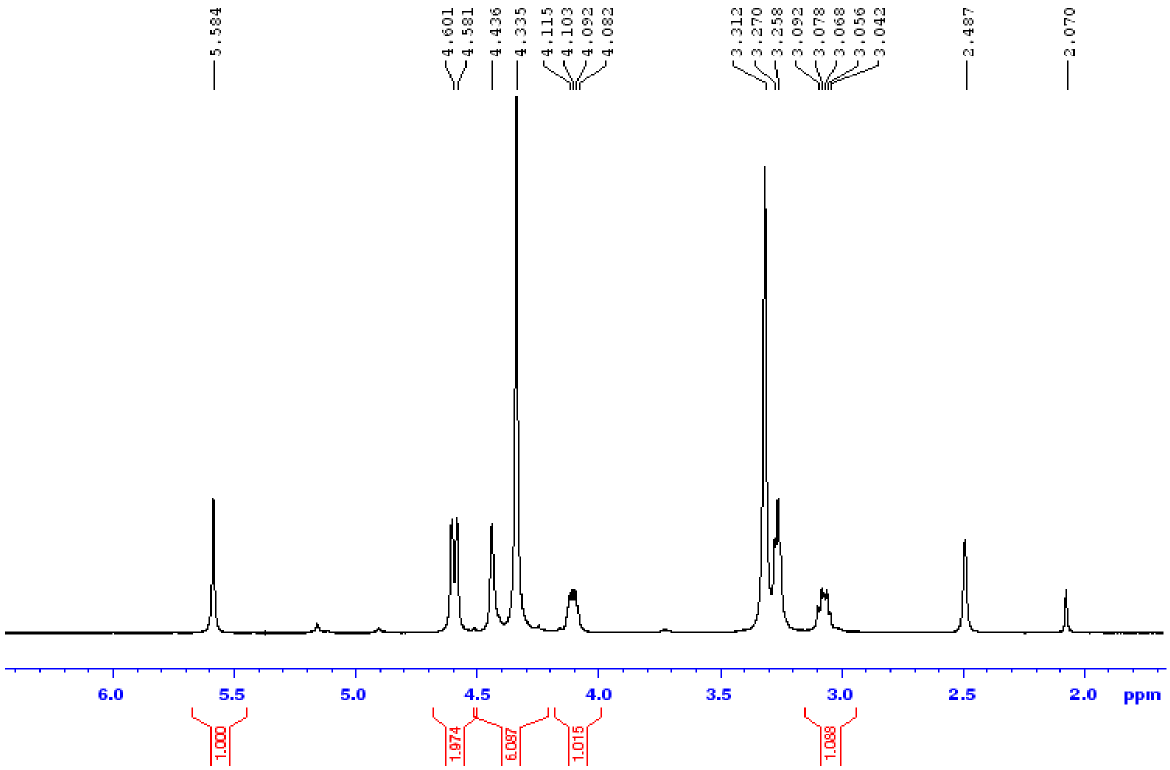

^{13}C NMR of **5**

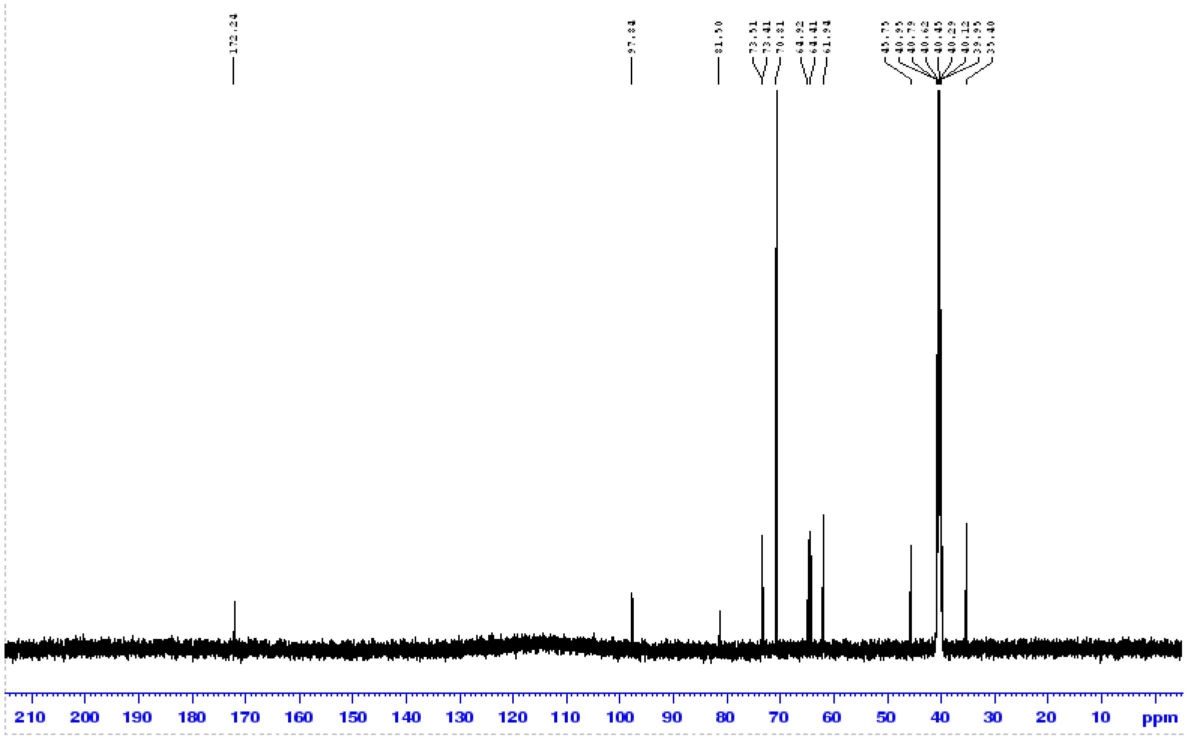

HMBC of **5**

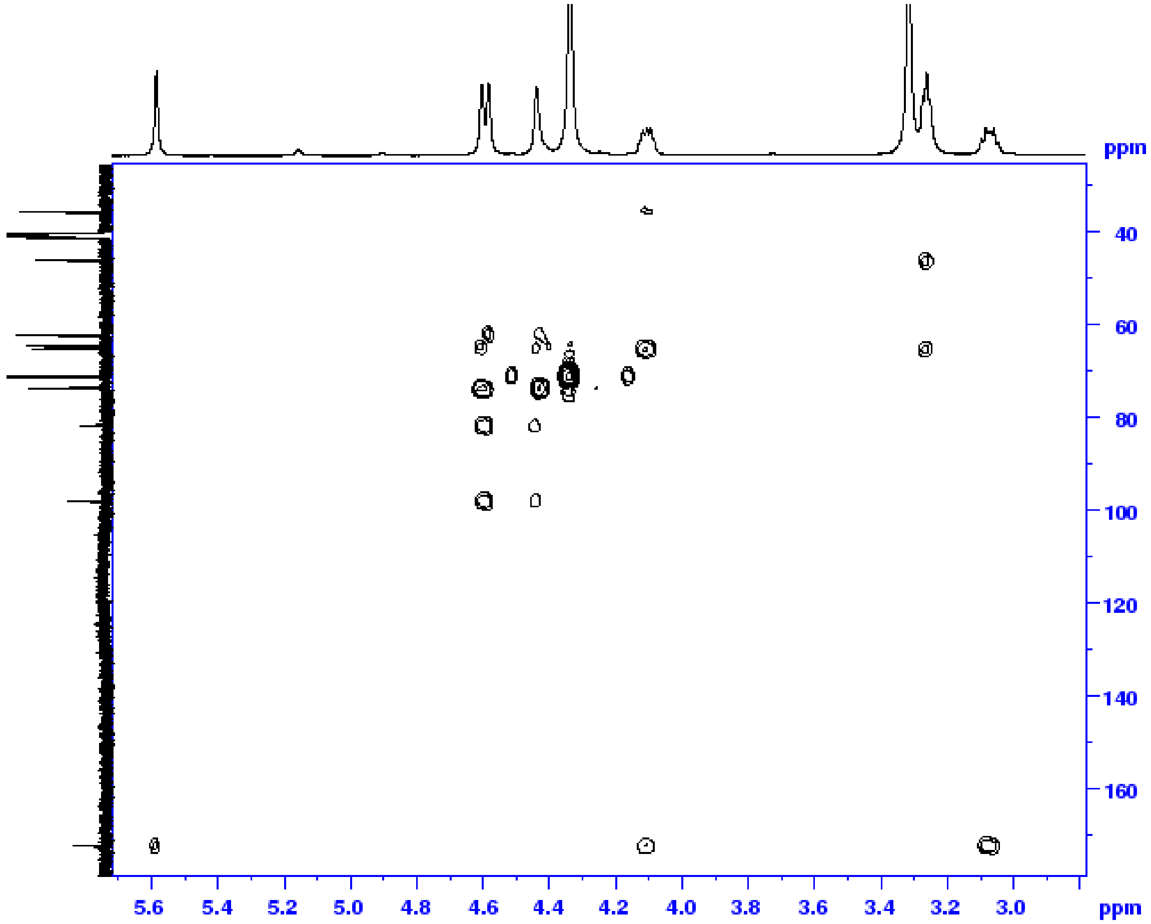

ppm
40
60
80
100
120
140
160
5.6
5.4
5.2
5.0
4.8
4.6
4.4
4.2
4.0
3.8
3.6
3.4
3.2
3.0
ppm

^{1}H NMR of **7**

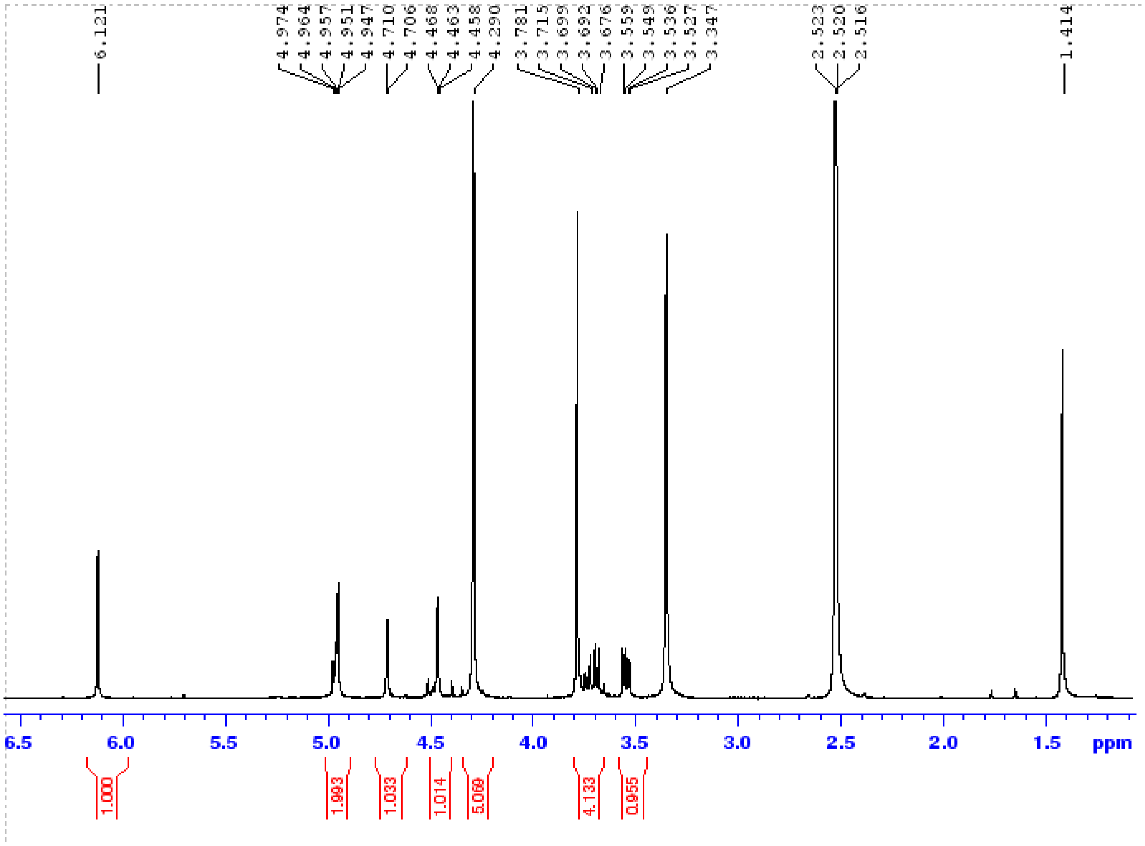

^{13}C NMR of **7**

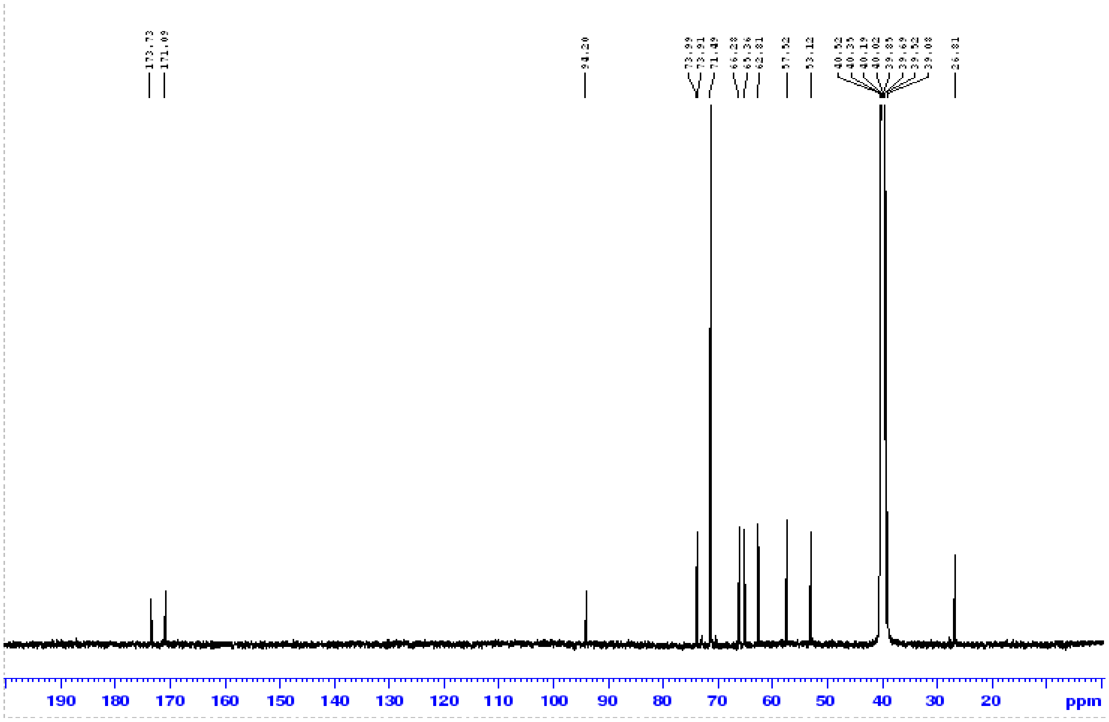

HSQC of **7**

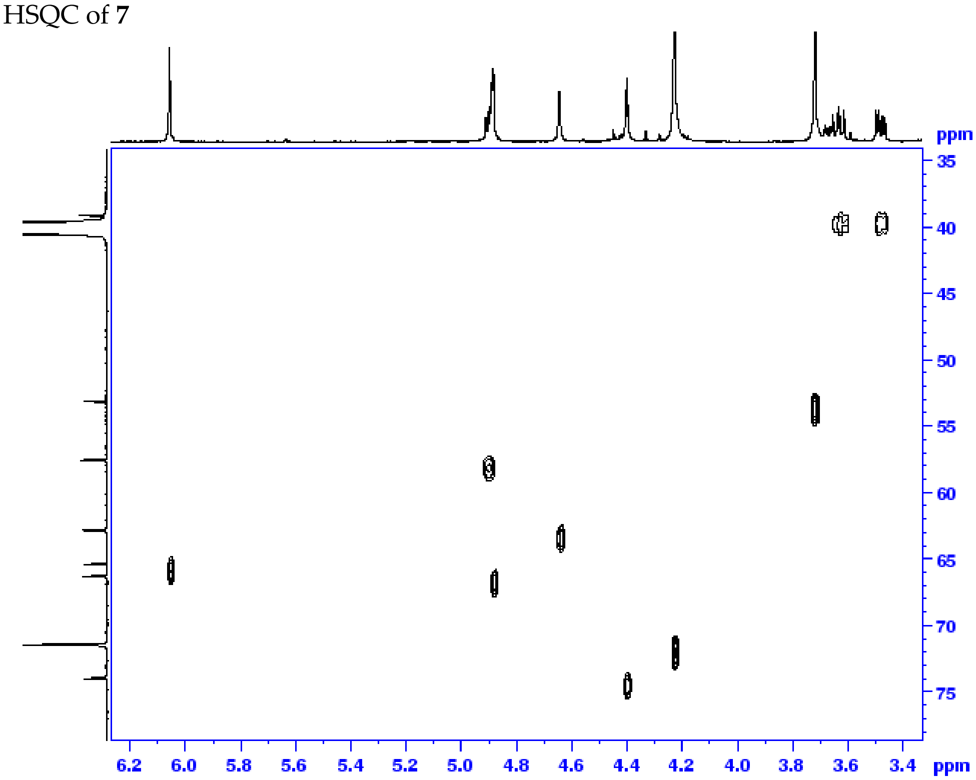

HMBC of **7**

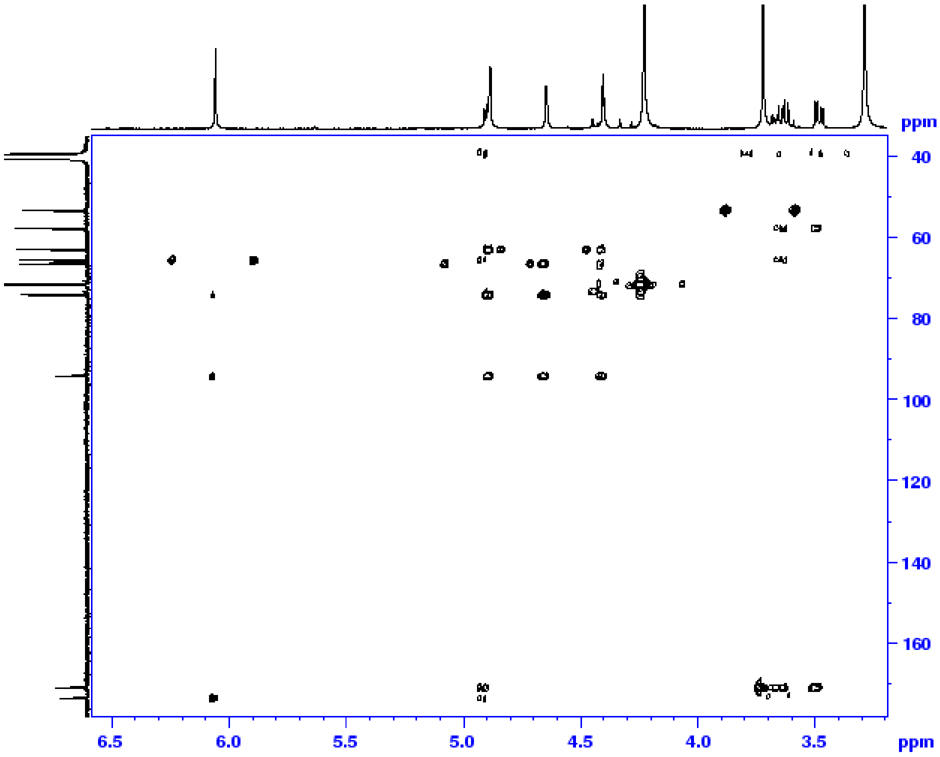

NOESY of **7**

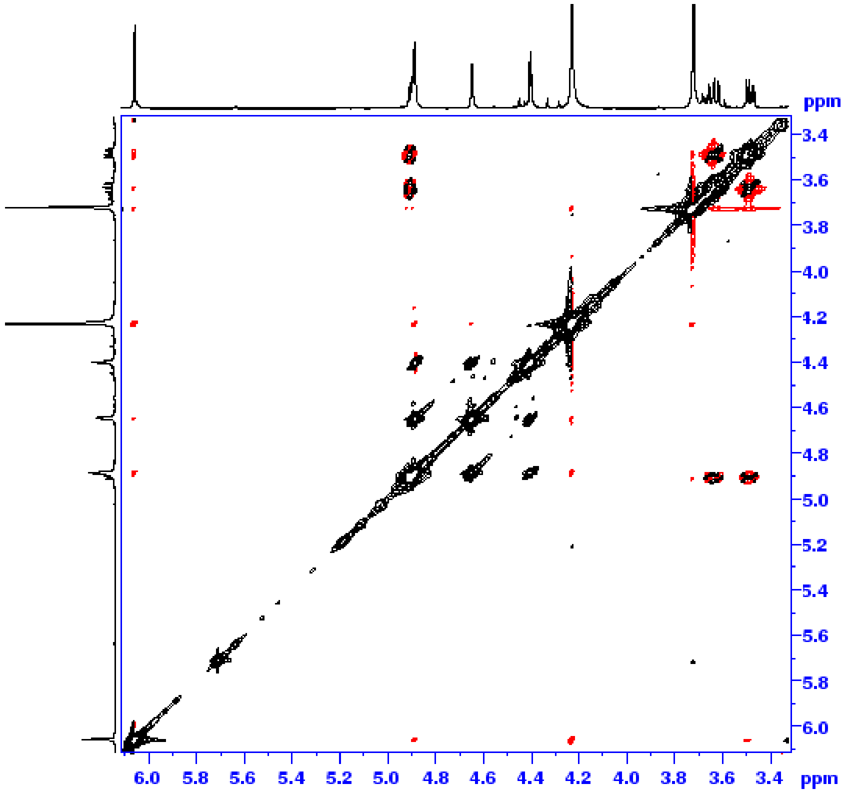

^{1}H NMR of **9**

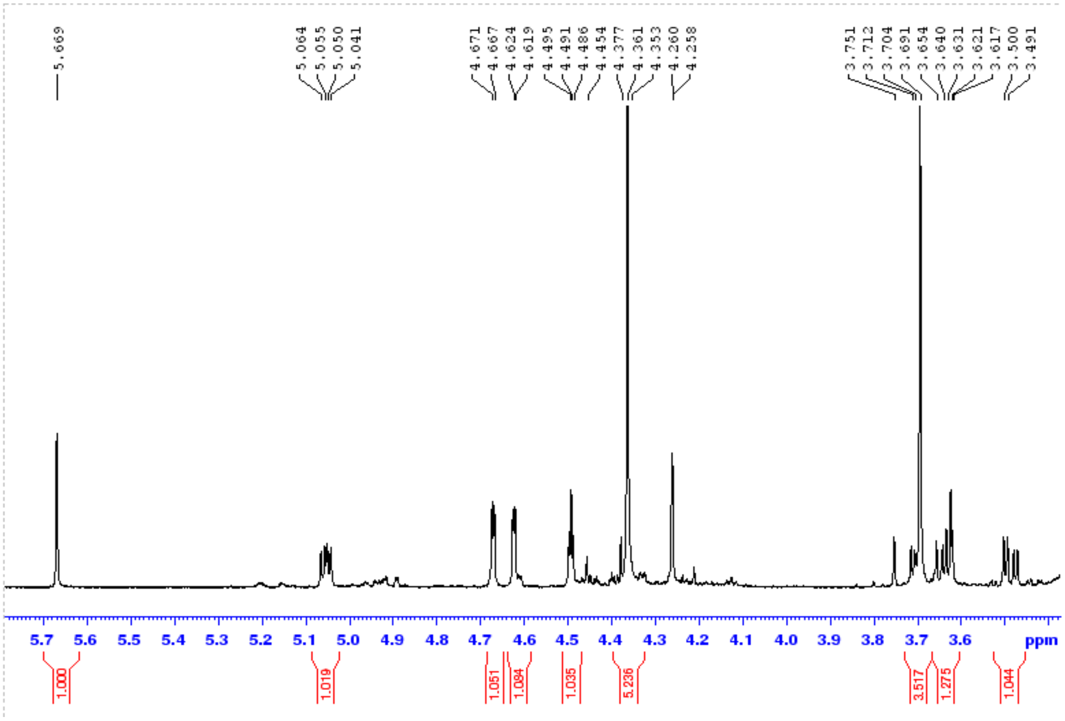

^{13}C NMR of **9**

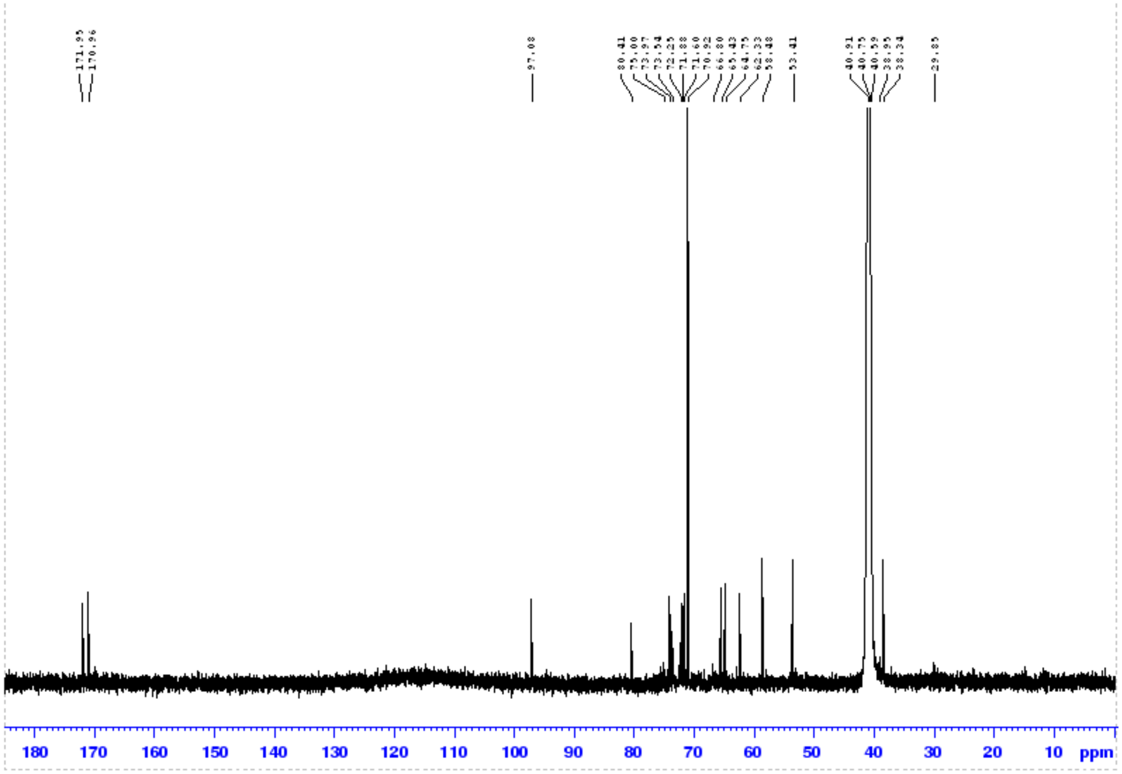

HMBC of **9**

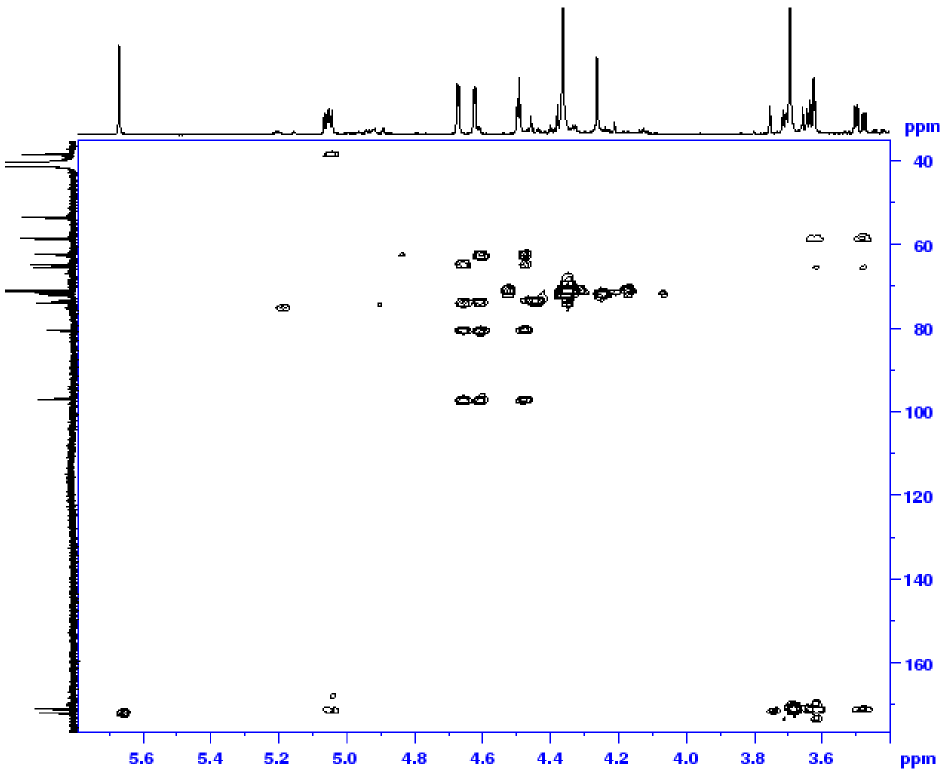

^{1}H NMR of **13**

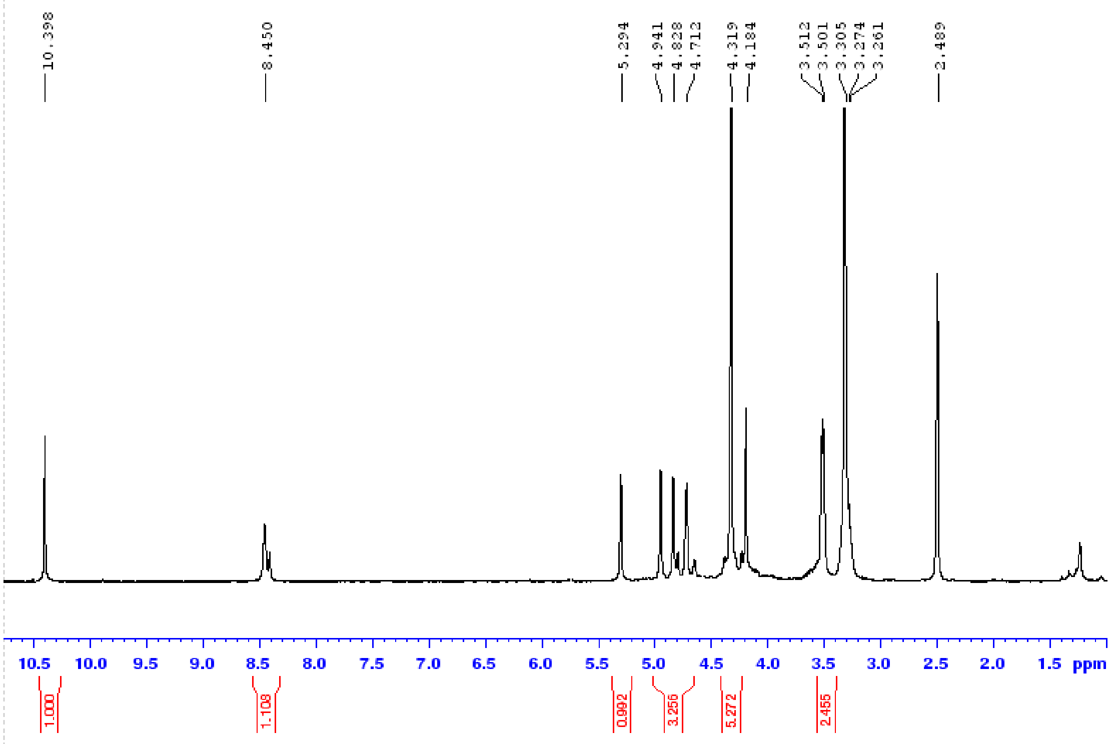

^{13}C NMR of **13**

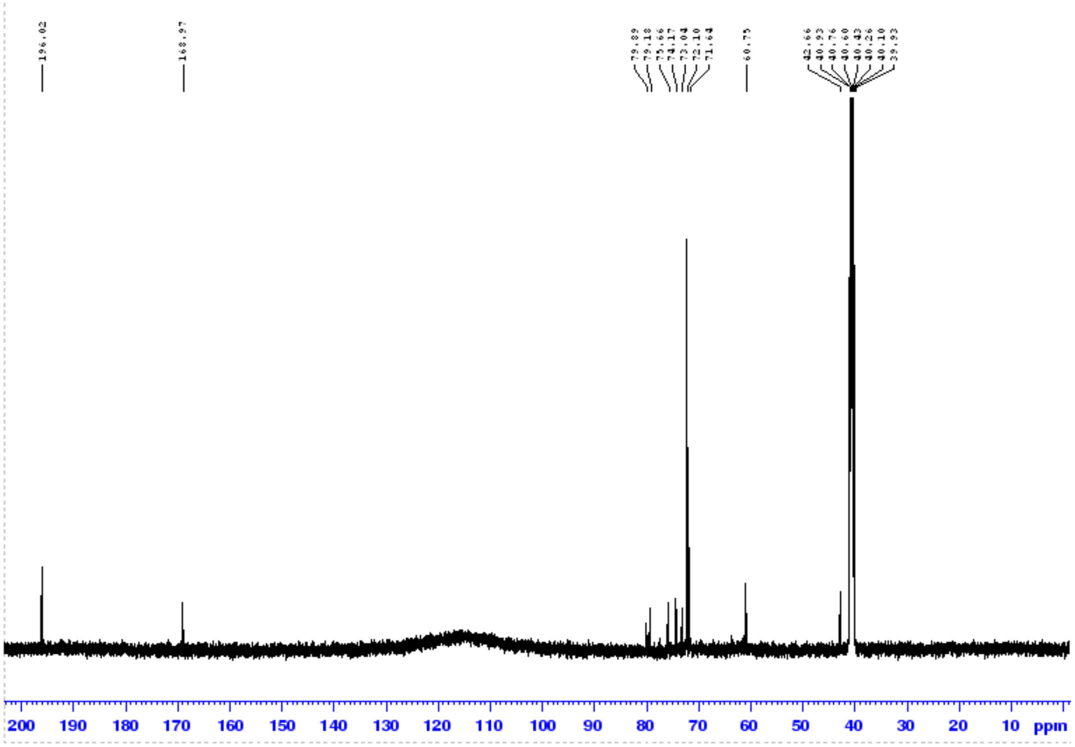

^{1}H NMR of **21**

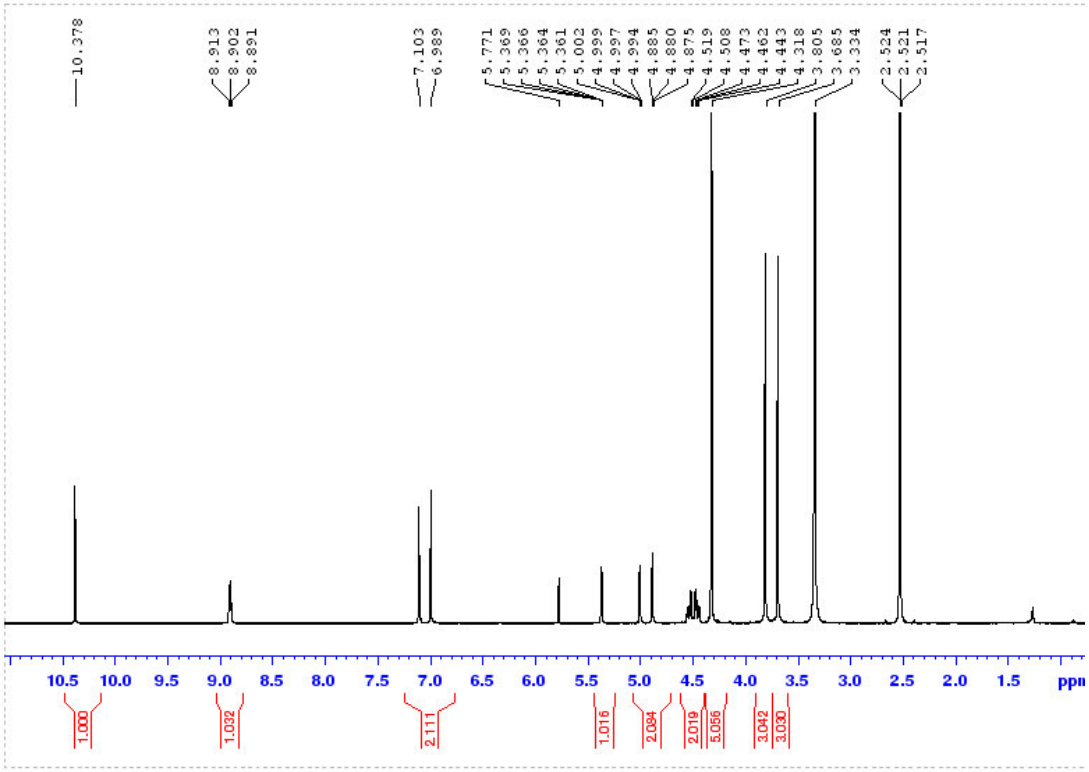

^{13}C NMR of **21**

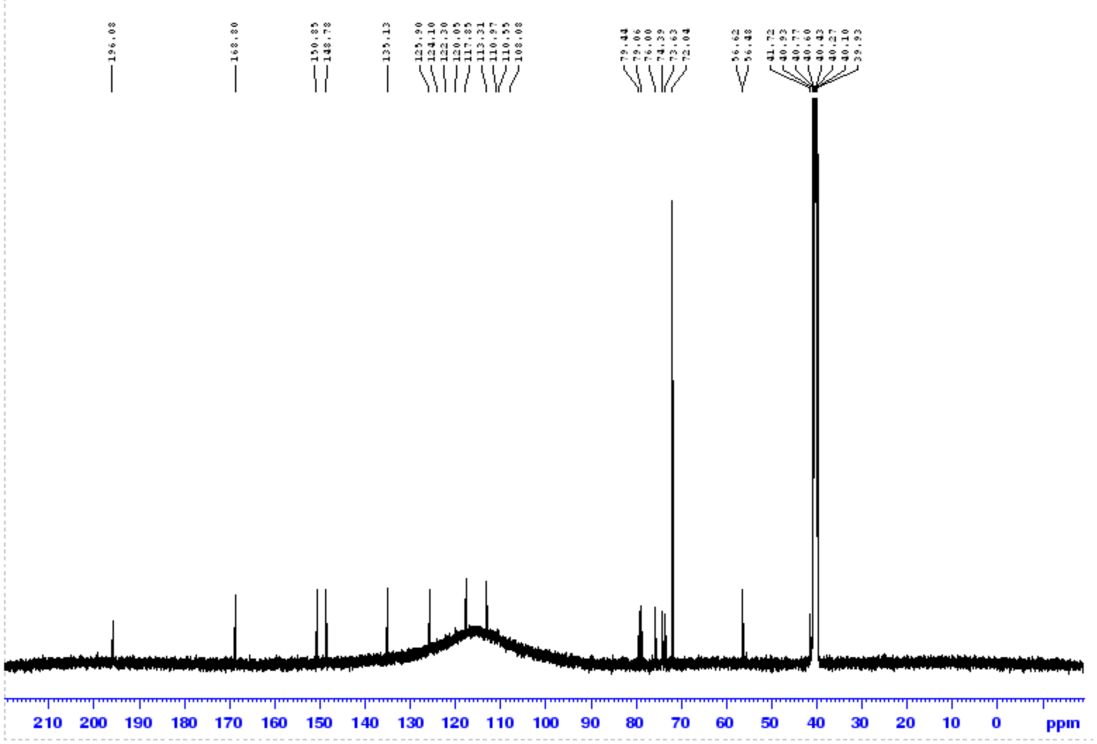

HSQC of **21**

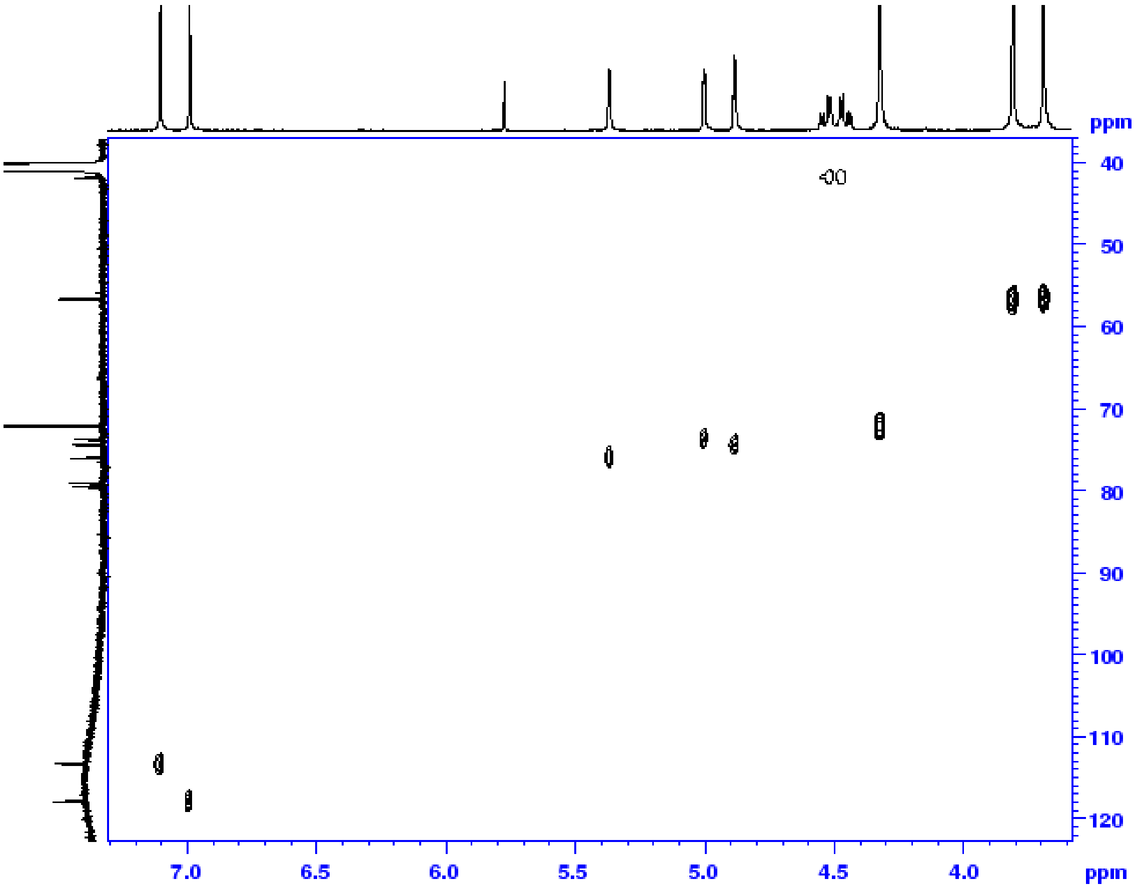

S.3.

Compound 2

Row	Highlight	Display	Tag	Symbol	X	Y	Z
1	No	Show	1	H	-0.6298850	2.3455640	1.5873610
2	No	Show	2	C	-0.1224730	1.3988920	1.4555250
3	No	Show	3	C	-0.3400710	0.2002210	2.1904310
4	No	Show	4	C	0.8671040	1.1470330	0.4547140
5	No	Show	5	C	0.4986310	-0.8179150	1.6579530
6	No	Show	6	H	-1.0604810	0.0673790	2.9880200
7	No	Show	7	C	1.2579490	-0.2458730	0.5828660
8	No	Show	8	Fe	-0.7141140	-0.0969530	0.1844080
9	No	Show	9	C	-1.8483860	-1.6710560	-0.4544010
10	No	Show	10	C	-1.1104450	-1.1195110	-1.5454210
11	No	Show	11	C	-2.6761770	-0.6378200	0.0825200
12	No	Show	12	H	-1.7637220	-2.6821840	-0.0760280
13	No	Show	13	C	-1.4810030	0.2522170	-1.6827850
14	No	Show	14	H	-0.3666260	-1.6386560	-2.1376120
15	No	Show	15	C	-2.4478000	0.5517390	-0.6757750
16	No	Show	16	H	-3.3291830	-0.7279940	0.9417870
17	No	Show	17	H	-1.0677260	0.9568520	-2.3937610
18	No	Show	18	H	-2.8949790	1.5209600	-0.4934620
19	No	Show	19	H	0.5472560	-1.8498740	1.9791930
20	No	Show	20	C	1.3027800	2.1347180	-0.5512460
21	No	Show	21	O	0.9014050	3.2874820	-0.5596610
22	No	Show	22	H	2.0224610	1.7701430	-1.3052980
23	No	Show	23	C	2.1975370	-0.9625820	-0.2749170
24	No	Show	24	O	2.8726220	-0.5434130	-1.1701600
25	No	Show	25	F	2.2590820	-2.2860380	0.0574420

Compound 4

Row	Highlight	Display	Tag	Symbol	X	Y	Z
1	No	Show	1	C	0.5680310	-1.8784840	0.6553990
2	No	Show	2	C	1.3892190	-1.3513020	1.7113800
3	No	Show	3	H	0.6688280	-2.8558660	0.2002590
4	No	Show	4	C	0.9754550	-0.0126160	2.0126110
5	No	Show	5	H	2.2227840	-1.8689430	2.1706750
6	No	Show	6	H	1.4269850	0.6540490	2.7357080
7	No	Show	7	C	-0.3704170	-0.8626530	0.3280410
8	No	Show	8	H	4.2093860	-0.9560670	-0.1966710
9	No	Show	9	C	3.4556850	-0.2973060	-0.6101450
10	No	Show	10	C	3.1648810	1.0297700	-0.1670710
11	No	Show	11	C	2.5427050	-0.6215080	-1.6607370
12	No	Show	12	C	2.0723480	1.5251280	-0.9412450
13	No	Show	13	H	3.6553750	1.5518240	0.6452010
14	No	Show	14	C	1.6875930	0.5049170	-1.8644440
15	No	Show	15	H	2.4835810	-1.5695130	-2.1813660

16	No	Show	16	H	0.8641070	0.5642280	-2.5656570
17	No	Show	17	Fe	1.5298850	-0.1734420	0.0540250
18	No	Show	18	H	1.5842120	2.4833270	-0.8116790
19	No	Show	19	C	-0.1209910	0.2731300	1.1474690
20	No	Show	20	C	-1.0989920	1.3294630	0.8072760
21	No	Show	21	O	-1.3237510	2.3990350	1.3501850
22	No	Show	22	N	-1.8073760	0.8347130	-0.2965610
23	No	Show	23	S	-3.1046480	-1.4386210	-0.1246460
24	No	Show	24	C	-4.0015050	-0.0260590	-0.8538860
25	No	Show	25	H	-5.0061730	0.0157130	-0.4281850
26	No	Show	26	H	-4.0930930	-0.1685840	-1.9346170
27	No	Show	27	C	-3.1884380	1.2430600	-0.5270260
28	No	Show	28	C	-1.5315900	-0.5734400	-0.5650010
29	No	Show	29	H	-1.3320410	-0.7489560	-1.6270060
30	No	Show	30	H	-3.5504860	1.7414310	0.3765000
31	No	Show	31	H	-3.2583430	1.9569120	-1.3547630

Compound 5

Row	Highlight		Display	Tag	Symbol X	Y	Z
1	No	Show	1	C	0.8834050	0.4005960	2.0455270
2	No	Show	2	C	1.8562070	1.3261860	1.5322630
3	No	Show	3	H	1.0317340	-0.2764260	2.8778110
4	No	Show	4	C	1.3106950	2.0049230	0.3935700
5	No	Show	5	H	2.8626980	1.4508090	1.9135770
6	No	Show	6	H	1.8140550	2.7382700	-0.2229760
7	No	Show	7	C	-0.2696520	0.5195810	1.2238160
8	No	Show	8	H	2.1671900	-2.5659260	0.8713630
9	No	Show	9	C	2.1011660	-1.9035110	0.0171800
10	No	Show	10	C	3.0884960	-0.9546340	-0.3903850
11	No	Show	11	C	0.9855020	-1.7867430	-0.8656410
12	No	Show	12	C	2.5797840	-0.2488990	-1.5241830
13	No	Show	13	H	4.0375260	-0.7724360	0.0988480
14	No	Show	14	C	1.2793390	-0.7615980	-1.8159720
15	No	Show	15	H	0.0595320	-2.3395230	-0.7814300
16	No	Show	16	H	0.6149860	-0.3928610	-2.5881570
17	No	Show	17	Fe	1.3475380	-0.0055550	0.0816610
18	No	Show	18	H	3.0716580	0.5655170	-2.0416280
19	No	Show	19	C	-0.0114100	1.4997510	0.2217970
20	No	Show	20	C	-1.2031080	1.6236220	-0.6453810
21	No	Show	21	O	-1.3547170	2.2073040	-1.7070770
22	No	Show	22	N	-2.2005300	0.8953410	0.0151150
23	No	Show	23	S	-2.2400960	-1.6290970	0.8691960
24	No	Show	24	C	-2.9984290	-1.2188430	-0.7447970
25	No	Show	25	H	-3.9009820	-1.8217260	-0.8696150
26	No	Show	26	H	-2.3039790	-1.4425490	-1.5593880
27	No	Show	27	C	-3.3076960	0.2809820	-0.6851010
28	No	Show	28	C	-1.7189380	0.1359190	1.1641040
29	No	Show	29	H	-2.2335460	0.4463050	2.0804250

30	No	Show	30	H	-4.2427620	0.4566040	-0.1425130
31	No	Show	31	H	-3.3832720	0.7341010	-1.6766370

Compound 7

Row	Highlight	Display	Tag	Symbol X	Y	Z	
1	No	Show	1	C	-2.1458120	1.7744900	-0.5528720
2	No	Show	2	C	-2.9880820	1.5803430	0.5958940
3	No	Show	3	H	-2.4515320	2.2028050	-1.4991800
4	No	Show	4	C	-2.2357260	0.9446320	1.6367600
5	No	Show	5	H	-4.0407850	1.8311210	0.6464190
6	No	Show	6	H	-2.6084520	0.6367250	2.6049620
7	No	Show	7	C	-0.8623050	1.2764420	-0.2016410
8	No	Show	8	H	-4.8467480	-0.9628040	-0.7780700
9	No	Show	9	C	-3.8283850	-1.3231880	-0.6993220
10	No	Show	10	C	-3.2356140	-1.9238270	0.4541120
11	No	Show	11	C	-2.8261080	-1.2370250	-1.7146710
12	No	Show	12	C	-1.8692740	-2.2060120	0.1521080
13	No	Show	13	H	-3.7235970	-2.0930530	1.4060690
14	No	Show	14	C	-1.6147440	-1.7813780	-1.1876060
15	No	Show	15	H	-2.9528010	-0.7972000	-2.6964940
16	No	Show	16	H	-0.6566350	-1.8295470	-1.6909770
17	No	Show	17	Fe	-2.2483620	-0.2084980	-0.0474920
18	No	Show	18	H	-1.1370240	-2.6156660	0.8371080
19	No	Show	19	C	-0.9154820	0.7676950	1.1273320
20	No	Show	20	C	0.4114160	0.2232590	1.4829390
21	No	Show	21	O	0.8411450	-0.1944340	2.5455240
22	No	Show	22	N	1.1658660	0.3142490	0.3040920
23	No	Show	23	S	1.5419670	2.6358150	-0.8277780
24	No	Show	24	C	3.0236830	1.6049020	-0.5688970
25	No	Show	25	H	3.8064870	2.2076790	-0.1060030
26	No	Show	26	H	3.3891010	1.2400680	-1.5328250
27	No	Show	27	C	2.6039490	0.4188100	0.3521150
28	No	Show	28	C	0.5181530	1.0949250	-0.7399220
29	No	Show	29	H	0.5656200	0.5895390	-1.7082110
30	No	Show	30	H	2.9060690	0.5977510	1.3877410
31	No	Show	31	C	3.2852460	-0.8555770	-0.1382090
32	No	Show	32	O	2.8110740	-1.6432530	-0.9271480
33	No	Show	33	O	4.5177110	-0.9580210	0.3908890
34	No	Show	34	C	5.2782720	-2.1020200	-0.0331410
35	No	Show	35	H	5.4207670	-2.0866950	-1.1157060
36	No	Show	36	H	6.2347650	-2.0229210	0.4812680
37	No	Show	37	H	4.7650850	-3.0228110	0.2504920

Compound 8

Row	Highlight	Display	Tag	Symbol X	Y	Z	
1	No	Show	1	C	2.2167760	0.5644650	-1.8195570
2	No	Show	2	C	2.7094250	-0.7860070	-1.8370830

3	No	Show	3	H	2.7958470	1.4521920	-2.0425370
4	No	Show	4	C	1.6633670	-1.6785580	-1.4333030
5	No	Show	5	H	3.7280610	-1.0760200	-2.0651770
6	No	Show	6	H	1.7378070	-2.7522140	-1.3197480
7	No	Show	7	C	0.8569440	0.4997970	-1.4117210
8	No	Show	8	H	3.8649230	1.5787230	1.0153790
9	No	Show	9	C	3.2620270	0.7265700	1.3043640
10	No	Show	10	C	3.6601440	-0.6441290	1.2430460
11	No	Show	11	C	1.9106290	0.7738140	1.7614070
12	No	Show	12	C	2.5515160	-1.4438040	1.6595940
13	No	Show	13	H	4.6188380	-1.0144380	0.9009390
14	No	Show	14	C	1.4700760	-0.5672290	1.9773650
15	No	Show	15	H	1.3123740	1.6692640	1.8635690
16	No	Show	16	H	0.4765290	-0.8745000	2.2810020
17	No	Show	17	Fe	2.0532810	-0.3200280	0.0314360
18	No	Show	18	H	2.5190840	-2.5260170	1.6849550
19	No	Show	19	C	0.5193880	-0.8654780	-1.1839800
20	No	Show	20	C	-0.8873290	-0.9500730	-0.7441700
21	No	Show	21	O	-1.5225180	-1.8996980	-0.3148470
22	No	Show	22	N	-1.3978600	0.3417470	-0.9330080
23	No	Show	23	S	-0.5374710	2.7372100	-0.1251380
24	No	Show	24	C	-1.8628240	1.9006390	0.8105880
25	No	Show	25	H	-2.5761340	2.6507150	1.1588320
26	No	Show	26	H	-1.4657160	1.3576140	1.6699590
27	No	Show	27	C	-2.5003200	0.9265420	-0.1827280
28	No	Show	28	C	-0.3922770	1.3252250	-1.3308810
29	No	Show	29	H	-0.6336410	1.7473200	-2.3131250
30	No	Show	30	C	-3.3837840	-0.1158160	0.5082430
31	No	Show	31	H	-3.1377650	1.4777890	-0.8839310
32	No	Show	32	O	-3.3621050	-0.3467720	1.6974700
33	No	Show	33	O	-4.2451830	-0.6520080	-0.3654180
34	No	Show	34	C	-5.0418850	-1.7305210	0.1519180
35	No	Show	35	H	-5.6584700	-2.0624220	-0.6821920
36	No	Show	36	H	-4.3893520	-2.5358980	0.4934830
37	No	Show	37	H	-5.6659240	-1.3837690	0.9782890

Compound 9

Row	Highlight		Display	Tag	Symbol X	Y	Z
1	No	Show	1	C	1.5692300	-1.0110270	-1.8675400
2	No	Show	2	C	2.4319070	-1.9050130	-1.1447270
3	No	Show	3	H	1.7576490	-0.6106470	-2.8561650
4	No	Show	4	C	1.8732640	-2.1613060	0.1494150
5	No	Show	5	H	3.3836530	-2.2796680	-1.5024020
6	No	Show	6	H	2.3078580	-2.7708220	0.9311270
7	No	Show	7	C	0.4696240	-0.7240960	-1.0154270
8	No	Show	8	H	4.3716710	1.0731700	-1.3950390
9	No	Show	9	C	3.7822010	1.0894300	-0.4863210

10	No	Show	10	C	4.0331260	0.3181270	0.6894460
11	No	Show	11	C	2.5870380	1.8413730	-0.2753000
12	No	Show	12	C	2.9910910	0.5893560	1.6270970
13	No	Show	13	H	4.8420370	-0.3895570	0.8249430
14	No	Show	14	C	2.0996460	1.5313930	1.0295150
15	No	Show	15	H	2.1012300	2.4886570	-0.9938730
16	No	Show	16	H	1.1913210	1.9135620	1.4764900
17	No	Show	17	Fe	2.2167770	-0.1687960	-0.1045730
18	No	Show	18	H	2.8678490	0.1250290	2.5978030
19	No	Show	19	C	0.6505600	-1.4312950	0.2092030
20	No	Show	20	C	-0.4865750	-1.1392200	1.1061850
21	No	Show	21	O	-0.6592720	-1.3953600	2.2868400
22	No	Show	22	N	-1.4030090	-0.4529800	0.2986240
23	No	Show	23	S	-1.1037870	1.7486110	-1.1572540
24	No	Show	24	C	-1.8855130	1.8768750	0.4933690
25	No	Show	25	H	-2.7142480	2.5847990	0.4493120
26	No	Show	26	H	-1.1574240	2.2164930	1.2334070
27	No	Show	27	C	-2.3715110	0.4601950	0.8423980
28	No	Show	28	C	-0.8930300	-0.0976290	-1.0192210
29	No	Show	29	H	-1.5173000	-0.5340760	-1.8059250
30	No	Show	30	H	-2.3925990	0.3152060	1.9290300
31	No	Show	31	C	-3.8057810	0.2392170	0.3573150
32	No	Show	32	O	-4.6925620	1.0426510	0.5589110
33	No	Show	33	O	-3.9744260	-0.9330910	-0.2676510
34	No	Show	34	C	-5.3208260	-1.2094280	-0.6909900
35	No	Show	35	H	-5.2805680	-2.1893950	-1.1639480
36	No	Show	36	H	-5.9932240	-1.2247090	0.1689290
37	No	Show	37	H	-5.6590750	-0.4515530	-1.4004100

Compound 10

Row	Highlight	Display	Tag	Symbol	X	Y	Z
1	No	Show	1	C	-1.7826170	1.4340790	1.3184670
2	No	Show	2	C	-2.2848150	0.3430250	2.1083690
3	No	Show	3	H	-2.2006190	2.4321170	1.2753410
4	No	Show	4	C	-1.4943410	-0.8259140	1.8631440
5	No	Show	5	H	-3.1572060	0.3877180	2.7493130
6	No	Show	6	H	-1.6496400	-1.8104290	2.2850600
7	No	Show	7	C	-0.6553170	0.9354590	0.6117400
8	No	Show	8	H	-4.8735390	0.9382670	-0.3321920
9	No	Show	9	C	-4.1603350	0.2130270	-0.7044580
10	No	Show	10	C	-4.0545060	-1.1494360	-0.2901130
11	No	Show	11	C	-3.1253240	0.4652860	-1.6566360
12	No	Show	12	C	-2.9527830	-1.7387180	-0.9817150
13	No	Show	13	H	-4.6695140	-1.6362790	0.4568510
14	No	Show	14	C	-2.3790260	-0.7402900	-1.8269770
15	No	Show	15	H	-2.9218070	1.4158810	-2.1353970
16	No	Show	16	H	-1.5005790	-0.8655520	-2.4488980
17	No	Show	17	Fe	-2.3281430	-0.1418000	0.1284520

18	No	Show	18	H	-2.5829670	-2.7483470	-0.8516660
19	No	Show	19	C	-0.4838090	-0.4392000	0.9360350
20	No	Show	20	C	0.6791720	-0.9734200	0.1967830
21	No	Show	21	O	1.2039160	-2.0740220	0.2307350
22	No	Show	22	N	1.1289020	0.0933490	-0.5904200
23	No	Show	23	S	1.6575780	2.5538490	0.1613340
24	No	Show	24	C	2.7854430	1.7699700	-1.0372710
25	No	Show	25	H	3.8168490	2.0153480	-0.7775280
26	No	Show	26	H	2.5839310	2.1483810	-2.0439400
27	No	Show	27	C	2.5171380	0.2425680	-0.9883460
28	No	Show	28	C	0.3889020	1.3397120	-0.3791160
29	No	Show	29	H	2.6989750	-0.1902770	-1.9762430
30	No	Show	30	C	3.4796860	-0.4203650	0.0038690
31	No	Show	31	O	3.4637770	-0.2621250	1.2022400
32	No	Show	32	O	4.4078620	-1.1333810	-0.6613150
33	No	Show	33	C	5.3789260	-1.7882660	0.1712860
34	No	Show	34	H	6.0340440	-2.3261300	-0.5126940
35	No	Show	35	H	5.9452350	-1.0552000	0.7499900
36	No	Show	36	H	4.8806500	-2.4806060	0.8522540
37	No	Show	37	H	-0.0384630	1.6980200	-1.3220520

Compound 11

Row	Highlight	Display	Tag	Symbol	X	Y	Z
1	No	Show	1	H	-2.6304330	1.4633150	1.7884440
2	No	Show	2	C	-1.6663910	1.0365580	1.5431890
3	No	Show	3	C	-1.0230660	-0.0501800	2.1951890
4	No	Show	4	C	-0.8569540	1.4697780	0.4517140
5	No	Show	5	C	0.2047530	-0.3179400	1.5324920
6	No	Show	6	H	-1.4246170	-0.6204100	3.0234390
7	No	Show	7	C	0.3456320	0.6402250	0.4559060
8	No	Show	8	Fe	-1.3225460	-0.4776890	0.1967220
9	No	Show	9	C	-1.4191670	-2.4315610	-0.4237630
10	No	Show	10	C	-1.2194890	-1.5680760	-1.5420900
11	No	Show	11	C	-2.6349210	-2.0424200	0.2157140
12	No	Show	12	H	-0.7580320	-3.2265760	-0.1018050
13	No	Show	13	C	-2.3063900	-0.6457030	-1.5946170
14	No	Show	14	H	-0.3718320	-1.5793860	-2.2165780
15	No	Show	15	C	-3.1824300	-0.9395280	-0.5068790
16	No	Show	16	H	-3.0506040	-2.4822290	1.1137660
17	No	Show	17	H	-2.4293990	0.1575960	-2.3102530
18	No	Show	18	H	-4.0814260	-0.3927640	-0.2510830
19	No	Show	19	H	0.8781430	-1.1327090	1.7686460
20	No	Show	20	C	-1.2332160	2.5635910	-0.4751520
21	No	Show	21	O	-2.2835310	3.1656330	-0.3640260
22	No	Show	22	H	-0.5105570	2.8034480	-1.2754260
23	No	Show	23	C	1.3784220	0.7088080	-0.5521090
24	No	Show	24	O	1.3379450	1.1799780	-1.6635600
25	No	Show	25	N	2.7086810	0.1410340	-0.1542330

26	No	Show	26	C	3.6000580	-0.2245840	-1.0825920
27	No	Show	27	C	3.3003750	0.0457920	1.0963830
28	No	Show	28	H	3.4246280	-0.2165800	-2.1488410
29	No	Show	29	C	4.5707790	-0.4048120	0.9059960
30	No	Show	30	H	2.7896230	0.3383650	2.0006360
31	No	Show	31	H	5.3658360	-0.6041430	1.6097990
32	No	Show	32	N	4.7250090	-0.5693970	-0.4579430
33	No	Show	33	H	5.5657320	-0.8940380	-0.9258850

Compound 13

Row	Highlight		Display	Tag	Symbol X	Y	Z
1	No	Show	1	H	-2.3706240	1.7522510	1.8102200
2	No	Show	2	C	-1.5002390	1.1563120	1.5685540
3	No	Show	3	C	-1.0652120	-0.0314070	2.2168250
4	No	Show	4	C	-0.6089370	1.4301490	0.4804090
5	No	Show	5	C	0.0991390	-0.5083050	1.5436250
6	No	Show	6	H	-1.5607110	-0.5231710	3.0450080
7	No	Show	7	C	0.3957550	0.3846970	0.4611530
8	No	Show	8	Fe	-1.4592600	-0.3908340	0.2319020
9	No	Show	9	C	-1.7621930	-2.2761690	-0.4888100
10	No	Show	10	C	-1.4781210	-1.3732860	-1.5586550
11	No	Show	11	C	-2.9285800	-1.8041620	0.1892910
12	No	Show	12	H	-1.1711960	-3.1403350	-0.2109300
13	No	Show	13	C	-2.4706550	-0.3471940	-1.5444330
14	No	Show	14	H	-0.6256660	-1.4154380	-2.2249570
15	No	Show	15	C	-3.3663410	-0.6104010	-0.4638460
16	No	Show	16	H	-3.3782840	-2.2499920	1.0680560
17	No	Show	17	H	-2.5025540	0.5133890	-2.2007390
18	No	Show	18	H	-4.2017450	0.0109020	-0.1658810
19	No	Show	19	H	0.6199630	-1.4304290	1.7735810
20	No	Show	20	C	-0.7768920	2.5491440	-0.4651440
21	No	Show	21	O	-1.6456710	3.3992450	-0.3344280
22	No	Show	22	H	-0.0653640	2.5722950	-1.3079830
23	No	Show	23	C	1.5077250	0.2662260	-0.5240460
24	No	Show	24	O	1.4628800	0.7830670	-1.6424440
25	No	Show	25	N	2.5750950	-0.4689450	-0.0930500
26	No	Show	26	H	2.6424320	-0.7018850	0.8889310
27	No	Show	27	C	3.7830600	-0.6277080	-0.8795030
28	No	Show	28	H	4.0083260	-1.6945170	-0.9946120
29	No	Show	29	H	3.5917010	-0.2048510	-1.8672290
30	No	Show	30	C	4.9671860	0.0650740	-0.2308750
31	No	Show	31	H	4.7669590	1.1417360	-0.1488220
32	No	Show	32	H	5.8594240	-0.0795340	-0.8563310
33	No	Show	33	O	5.1452460	-0.5217890	1.0585890
34	No	Show	34	H	5.8286260	-0.0281150	1.5325980

Compound 14

Row	Highlight	Display	Tag	Symbol	X	Y	Z
1	No	Show	1	C	0.4784850	-1.8276070	0.8569710
2	No	Show	2	C	1.2367840	-1.1334120	1.8617300
3	No	Show	3	H	0.6514820	-2.8411170	0.5177330
4	No	Show	4	C	0.7300470	0.1999160	2.0046100
5	No	Show	5	H	2.0896270	-1.5398860	2.3921120
6	No	Show	6	H	1.1202210	0.9701490	2.6571650
7	No	Show	7	C	-0.5132700	-0.9191170	0.4010100
8	No	Show	8	H	4.0972810	-0.6391270	0.0553050
9	No	Show	9	C	3.3003210	-0.1408470	-0.4828780
10	No	Show	10	C	2.8457350	1.1960130	-0.2627590
11	No	Show	11	C	2.4846210	-0.7219070	-1.5020780
12	No	Show	12	C	1.7491140	1.4408060	-1.1438040
13	No	Show	13	H	3.2339320	1.8875990	0.4747610
14	No	Show	14	C	1.5255720	0.2560470	-1.9097660
15	No	Show	15	H	2.5547230	-1.7382110	-1.8700960
16	No	Show	16	H	0.7420390	0.1150950	-2.6443220
17	No	Show	17	Fe	1.3404750	-0.1386440	0.0841960
18	No	Show	18	H	1.1552330	2.3456680	-1.1833230
19	No	Show	19	C	-0.3599440	0.3151480	1.0933880
20	No	Show	20	C	-1.4071360	1.2555030	0.6302610
21	No	Show	21	O	-1.7425420	2.3365740	1.0828060
22	No	Show	22	N	-2.0261430	0.6169660	-0.4570570
23	No	Show	23	C	-3.9137250	-0.7526540	-0.6887220
24	No	Show	24	H	-4.7955470	-0.9525160	-0.0777670
25	No	Show	25	H	-4.1133100	-1.0795070	-1.7186930
26	No	Show	26	C	-3.4666630	0.7311770	-0.6589860
27	No	Show	27	C	-1.6605570	-0.8009460	-0.5521520
28	No	Show	28	H	-1.4146280	-1.0730140	-1.5891290
29	No	Show	29	H	-3.9011500	1.2971960	0.1691260
30	No	Show	30	H	-3.7101020	1.2449710	-1.5936250
31	No	Show	31	O	-2.8268880	-1.4990790	-0.1402930

Compound 15

Row	Highlight	Display	Tag	Symbol	X	Y	Z
1	No	Show	1	C	0.7676320	-0.9485250	-1.8662910
2	No	Show	2	C	1.6051690	-1.7940490	-1.0603860
3	No	Show	3	H	1.0273380	-0.5373940	-2.8340160
4	No	Show	4	C	0.9408380	-2.0791710	0.1776650
5	No	Show	5	H	2.6041880	-2.1196870	-1.3249770
6	No	Show	6	H	1.3306460	-2.6676200	0.9980930
7	No	Show	7	C	-0.4193960	-0.7159490	-1.1195550
8	No	Show	8	H	2.6224090	1.8724700	-1.4839680
9	No	Show	9	C	2.3016340	1.5919630	-0.4882380
10	No	Show	10	C	3.0076090	0.7356770	0.4112630
11	No	Show	11	C	1.0693670	1.9643270	0.1294920

12	No	Show	12	C	2.2104200	0.5765150	1.5860800
13	No	Show	13	H	3.9597860	0.2567260	0.2186020
14	No	Show	14	C	1.0134750	1.3359950	1.4116490
15	No	Show	15	H	0.2930780	2.5627580	-0.3289930
16	No	Show	16	H	0.1899480	1.3794660	2.1140690
17	No	Show	17	Fe	1.1931080	-0.0680200	-0.0620310
18	No	Show	18	H	2.4488450	-0.0445010	2.4407160
19	No	Show	19	C	-0.3195910	-1.4177150	0.1169450
20	No	Show	20	C	-1.5467550	-1.1723430	0.9086700
21	No	Show	21	O	-1.7948560	-1.4130360	2.0782550
22	No	Show	22	N	-2.4344080	-0.5598750	0.0101360
23	No	Show	23	C	-2.6648710	1.7327870	-0.1166860
24	No	Show	24	H	-3.3611190	2.5159900	-0.4282580
25	No	Show	25	H	-1.9710960	2.1459910	0.6259290
26	No	Show	26	C	-3.3572010	0.4779700	0.4293760
27	No	Show	27	C	-1.7992470	-0.1244500	-1.2334650
28	No	Show	28	H	-2.3291260	-0.5226640	-2.1089580
29	No	Show	29	H	-4.3409300	0.3243440	-0.0263680
30	No	Show	30	O	-1.9361950	1.2978540	-1.2726440
31	No	Show	31	H	-3.4600130	0.4650100	1.5159670

Compound 16

Row	Highlight		Display	Tag	Symbol X	Y	Z
1	No	Show	1	H	-2.3901860	2.1938770	1.4908430
2	No	Show	2	C	-1.6084640	1.4564100	1.3635940
3	No	Show	3	C	-1.3389870	0.3380740	2.1982810
4	No	Show	4	C	-0.6873340	1.4351830	0.2665180
5	No	Show	5	C	-0.2484530	-0.3895870	1.6345150
6	No	Show	6	H	-1.8974230	0.0526550	3.0814000
7	No	Show	7	C	0.1671350	0.2752170	0.4328210
8	No	Show	8	Fe	-1.7740870	-0.2728610	0.2844950
9	No	Show	9	C	-2.3594440	-2.1881760	-0.1138920
10	No	Show	10	C	-1.9236300	-1.5416860	-1.3105050
11	No	Show	11	C	-3.4458930	-1.4323660	0.4254450
12	No	Show	12	H	-1.9168240	-3.0712440	0.3305420
13	No	Show	13	C	-2.7418560	-0.3896590	-1.5122150
14	No	Show	14	H	-1.0823030	-1.8321940	-1.9271040
15	No	Show	15	C	-3.6821770	-0.3195160	-0.4397360
16	No	Show	16	H	-3.9708320	-1.6429040	1.3491450
17	No	Show	17	H	-2.6294440	0.3362740	-2.3075970
18	No	Show	18	H	-4.4133360	0.4645360	-0.2868580
19	No	Show	19	H	0.1427620	-1.3259150	2.0152950
20	No	Show	20	C	-0.6961260	2.4106880	-0.8400120
21	No	Show	21	O	-1.4483890	3.3734060	-0.8642250
22	No	Show	22	H	0.0239260	2.2192380	-1.6541580
23	No	Show	23	C	1.2541650	-0.1380510	-0.4965400
24	No	Show	24	O	1.2719260	0.1724500	-1.6888800

25	No	Show	25	N	2.2413810	-0.8976210	0.0680420
26	No	Show	26	H	2.2842580	-0.9662980	1.0753880
27	No	Show	27	C	3.3978990	-1.3305990	-0.6944040
28	No	Show	28	H	3.7997700	-2.2290100	-0.2175240
29	No	Show	29	H	3.0519840	-1.5988520	-1.6968730
30	No	Show	30	C	4.4943170	-0.2727430	-0.8206060
31	No	Show	31	H	4.1244140	0.6039710	-1.3552260
32	No	Show	32	H	5.3257020	-0.6842970	-1.4005320
33	No	Show	33	S	5.2283220	0.2441890	0.7727700
34	No	Show	34	H	4.2557610	1.1048960	1.1310540

Compound 19

Row	Highlight	Display	Tag	Symbol	X	Y	Z
1	No	Show	1	C	-2.6796890	1.9929390	-0.0940610
2	No	Show	2	C	-3.7828240	1.8315170	0.8131770
3	No	Show	3	H	-2.6025450	2.7392000	-0.8748370
4	No	Show	4	C	-3.5343300	0.7077800	1.6681690
5	No	Show	5	H	-4.6827020	2.4348250	0.8148150
6	No	Show	6	H	-4.1979670	0.3172190	2.4286180
7	No	Show	7	C	-1.7440830	0.9700490	0.2223020
8	No	Show	8	H	-6.1123280	0.5270640	-1.3097780
9	No	Show	9	C	-5.2579000	-0.1377240	-1.3411050
10	No	Show	10	C	-5.0203790	-1.2426500	-0.4666040
11	No	Show	11	C	-4.1458160	-0.0330870	-2.2319450
12	No	Show	12	C	-3.7622610	-1.8204690	-0.8157020
13	No	Show	13	H	-5.6587200	-1.5591230	0.3489130
14	No	Show	14	C	-3.2218280	-1.0736830	-1.9072850
15	No	Show	15	H	-4.0086990	0.7261440	-2.9922490
16	No	Show	16	H	-2.2639790	-1.2452310	-2.3834370
17	No	Show	17	Fe	-3.5289650	0.1395860	-0.2932890
18	No	Show	18	H	-3.2794210	-2.6436980	-0.3034750
19	No	Show	19	C	-2.2601960	0.1917170	1.2942670
20	No	Show	20	C	-1.2960120	-0.8873780	1.6093670
21	No	Show	21	O	-1.3419130	-1.7677750	2.4559050
22	No	Show	22	N	-0.2565420	-0.7039780	0.7033700
23	No	Show	23	S	0.8984740	1.7143250	0.3018060
24	No	Show	24	C	-0.3833000	0.4646980	-0.1391700
25	No	Show	25	H	-0.2483070	0.2138600	-1.1957510
26	No	Show	26	C	0.9374020	-1.5150850	0.7073800
27	No	Show	27	H	1.0078750	-1.9603830	1.7055450
28	No	Show	28	H	0.8317110	-2.3509300	0.0003550
29	No	Show	29	C	2.3009680	0.6260190	0.2002680
30	No	Show	30	C	2.2098070	-0.7570570	0.3828550
31	No	Show	31	C	3.5458140	1.2279090	-0.0529330
32	No	Show	32	C	3.3886940	-1.5088150	0.2699560
33	No	Show	33	C	4.7121680	0.4712430	-0.1162450
34	No	Show	34	H	3.5806670	2.3028190	-0.1986080

35	No	Show	35	C	4.6260830	-0.9320750	0.0322590
36	No	Show	36	H	3.3577870	-2.5893120	0.3969230
37	No	Show	37	O	5.9563790	0.9837550	-0.3239440
38	No	Show	38	O	5.7430130	-1.7273560	0.0161480
39	No	Show	39	C	6.0882750	2.3941890	-0.4009990
40	No	Show	40	H	5.5515020	2.8029910	-1.2655930
41	No	Show	41	H	5.7312830	2.8782900	0.5154960
42	No	Show	42	H	7.1548810	2.5872170	-0.5177190
43	No	Show	43	C	6.3684240	-1.8752940	-1.2593260
44	No	Show	44	H	7.2153560	-2.5461830	-1.1053600
45	No	Show	45	H	5.6748190	-2.3264400	-1.9798330
46	No	Show	46	H	6.7281410	-0.9163670	-1.6436380

Compound 20

Row	Highlight	Display	Tag	Symbol	X	Y	Z
1	No	Show	4	C	3.7002280	1.3799610	1.1901640
2	No	Show	2	C	3.8801280	0.2083120	1.9978910
3	No	Show	13	H	4.4503680	2.1248630	0.9585630
4	No	Show	5	C	2.6555520	-0.5429140	2.0257350
5	No	Show	6	H	4.8081570	-0.0917490	2.4696870
6	No	Show	14	H	2.5024560	-1.4898650	2.5282270
7	No	Show	3	C	2.3514430	1.3498910	0.7327950
8	No	Show	16	H	5.7667640	-1.7111700	-0.0047610
9	No	Show	8	C	4.8992490	-1.4346160	-0.5914590
10	No	Show	10	C	3.6761010	-2.1676770	-0.6796740
11	No	Show	7	C	4.7567460	-0.2447800	-1.3700940
12	No	Show	17	C	2.7809960	-1.4331860	-1.5153020
13	No	Show	19	H	3.4533330	-3.0980040	-0.1716310
14	No	Show	9	C	3.4466800	-0.2428850	-1.9378490
15	No	Show	15	H	5.4954370	0.5402940	-1.4744470
16	No	Show	18	H	3.0173620	0.5500130	-2.5378100
17	No	Show	1	Fe	3.3501950	-0.3062060	0.1035050
18	No	Show	23	H	1.7612300	-1.7058690	-1.7518870
19	No	Show	12	C	1.7168990	0.1814320	1.2413450
20	No	Show	11	C	1.3740500	2.1061580	-0.0841790
21	No	Show	21	O	1.5225850	3.0960670	-0.7875140
22	No	Show	20	N	0.1689130	1.4458610	0.1073240
23	No	Show	25	S	-0.4533580	-1.2116590	0.0463300
24	No	Show	22	C	0.2565800	0.2423650	0.9169760
25	No	Show	26	H	-0.3422510	0.3526230	1.8293160
26	No	Show	24	C	-0.9922320	1.7432820	-0.7199400
27	No	Show	29	H	-0.7114760	1.6075570	-1.7744330
28	No	Show	28	H	-1.2482740	2.7995140	-0.6001540
29	No	Show	30	C	-2.0686570	-0.4881980	-0.1216010
30	No	Show	27	C	-2.1893350	0.8760090	-0.4127450
31	No	Show	32	C	-3.2047210	-1.2906960	0.0448760
32	No	Show	31	C	-3.4786350	1.4128790	-0.5049240

33	No	Show	35	C	-4.4828260	-0.7500940	-0.0973360
34	No	Show	36	H	-3.0711270	-2.3391780	0.2897450
35	No	Show	33	C	-4.6182510	0.6314700	-0.3606070
36	No	Show	34	H	-3.6205470	2.4692770	-0.7213900
37	No	Show	38	O	-5.6379710	-1.4614030	0.0137200
38	No	Show	37	O	-5.8471430	1.2103860	-0.5418900
39	No	Show	40	C	-5.5394140	-2.8605520	0.2272170
40	No	Show	45	H	-5.0574530	-3.0888880	1.1854390
41	No	Show	44	H	-4.9917570	-3.3509470	-0.5860180
42	No	Show	46	H	-6.5644550	-3.2312300	0.2444950
43	No	Show	39	C	-6.6502280	1.3268510	0.6340580
44	No	Show	43	H	-7.5691820	1.8270020	0.3238440
45	No	Show	42	H	-6.1420520	1.9382300	1.3898320
46	No	Show	41	H	-6.8921480	0.3459430	1.0530840

Compound 18

Row	Highlight	Display	Tag	Symbol	X	Y	Z
1	No	Show	1	H	-4.1523440	0.5154290	2.8001640
2	No	Show	2	C	-3.4326540	0.0407550	2.1459540
3	No	Show	3	C	-3.2192380	-1.3536410	1.9734280
4	No	Show	4	C	-2.5537430	0.7471020	1.2621540
5	No	Show	5	C	-2.2054180	-1.5332690	0.9858420
6	No	Show	6	H	-3.7661420	-2.1477710	2.4667400
7	No	Show	7	C	-1.7826830	-0.2400060	0.5313610
8	No	Show	8	Fe	-3.7629620	-0.4748370	0.1947240
9	No	Show	9	C	-4.4932780	-1.5589370	-1.3751880
10	No	Show	10	C	-4.0672690	-0.2690840	-1.8158790
11	No	Show	11	C	-5.5013160	-1.3744180	-0.3788510
12	No	Show	12	H	-4.0932830	-2.5092450	-1.7072730
13	No	Show	13	C	-4.8135090	0.7114280	-1.0957330
14	No	Show	14	H	-3.2750000	-0.0610330	-2.5241720
15	No	Show	15	C	-5.6987490	0.0306380	-0.2058060
16	No	Show	16	H	-6.0001190	-2.1603000	0.1750270
17	No	Show	17	H	-4.6894100	1.7844820	-1.1698850
18	No	Show	18	H	-6.3677120	0.4985350	0.5056680
19	No	Show	19	H	-1.8686790	-2.4872610	0.5967420
20	No	Show	20	C	-2.5278920	2.2143810	1.1159270
21	No	Show	21	O	-3.2152600	2.9582910	1.7989070
22	No	Show	22	H	-1.8439260	2.6078430	0.3441920
23	No	Show	23	C	-0.7549740	0.0508050	-0.5029300
24	No	Show	24	O	-0.7912740	1.0681870	-1.2080480
25	No	Show	25	N	0.2230890	-0.8875020	-0.6171310
26	No	Show	26	H	0.2975070	-1.5753790	0.1209240
27	No	Show	27	C	1.3405260	-0.7912090	-1.5569550
28	No	Show	28	H	1.4531730	-1.7702860	-2.0344960
29	No	Show	29	H	1.0460230	-0.0836360	-2.3339500
30	No	Show	30	C	2.6491840	-0.4003070	-0.9094260
31	No	Show	31	C	3.0966870	0.9261500	-0.7950210

32	No	Show	32	C	3.4614010	-1.4158230	-0.3840900
33	No	Show	33	C	4.3192300	1.1928380	-0.1567430
34	No	Show	34	C	4.6741220	-1.1661590	0.2420930
35	No	Show	35	H	3.1521410	-2.4566040	-0.4643330
36	No	Show	36	C	5.1099360	0.1704640	0.3662520
37	No	Show	37	H	4.6310090	2.2267810	-0.0550770
38	No	Show	38	S	2.2749300	2.3474140	-1.4807150
39	No	Show	39	H	0.9958040	1.8968490	-1.4145490
40	No	Show	40	O	6.2894050	0.3655480	1.0161420
41	No	Show	41	O	5.3773010	-2.2144420	0.7798400
42	No	Show	42	C	6.7305860	1.7004690	1.2138160
43	No	Show	43	H	6.0087930	2.2757110	1.8048780
44	No	Show	44	H	7.6672370	1.6239080	1.7663700
45	No	Show	45	H	6.9143620	2.2093390	0.2602970
46	No	Show	46	C	6.6168840	-2.5129210	0.1351160
47	No	Show	47	H	7.3192400	-1.6780260	0.2105540
48	No	Show	48	H	7.0264970	-3.3813230	0.6540910
49	No	Show	49	H	6.4529670	-2.7645720	-0.9200170

Compound 22

Row	Highlight	Display	Tag	Symbol	X	Y	Z
1	No	Show	1	H	-2.8961410	1.5007170	1.6587110
2	No	Show	2	C	-1.9037520	1.1248900	1.4449670
3	No	Show	3	C	-1.1753010	0.1629010	2.1962270
4	No	Show	4	C	-1.1365950	1.5046890	0.3047070
5	No	Show	5	C	0.0638930	-0.0800690	1.5470650
6	No	Show	6	H	-1.5263440	-0.3460100	3.0851490
7	No	Show	7	C	0.1253310	0.7724220	0.3760690
8	No	Show	8	Fe	-1.4530690	-0.4912740	0.2550260
9	No	Show	9	C	-1.4056630	-2.4998530	-0.1644910
10	No	Show	10	C	-1.2896340	-1.7414460	-1.3679730
11	No	Show	11	C	-2.6394330	-2.1417230	0.4579790
12	No	Show	12	H	-0.6803730	-3.2052530	0.2219760
13	No	Show	13	C	-2.4449250	-0.9138240	-1.4892370
14	No	Show	14	H	-0.4537360	-1.7578840	-2.0566880
15	No	Show	15	C	-3.2805300	-1.1621600	-0.3591590
16	No	Show	16	H	-3.0075990	-2.5173870	1.4045970
17	No	Show	17	H	-2.6381750	-0.1978870	-2.2781540
18	No	Show	18	H	-4.2161790	-0.6628300	-0.1406940
19	No	Show	19	H	0.8065940	-0.8018270	1.8654120
20	No	Show	20	C	-1.6032120	2.4687180	-0.7211470
21	No	Show	21	O	-2.6975090	2.9935110	-0.6539040
22	No	Show	22	H	-0.9069550	2.6857040	-1.5506710
23	No	Show	23	C	1.1309620	0.7931960	-0.6591680
24	No	Show	24	O	1.0613320	1.1765210	-1.7988320
25	No	Show	25	C	3.0495520	0.5824640	0.9533320
26	No	Show	26	C	3.2134760	-0.4008200	-1.1826690
27	No	Show	27	C	4.3443230	0.2005490	1.2565800

28	No	Show	28	H	2.4309860	1.1600170	1.6304070
29	No	Show	29	C	4.5031910	-0.8223660	-0.9207600
30	No	Show	30	H	2.7103560	-0.5566800	-2.1303600
31	No	Show	31	C	5.0801380	-0.5190650	0.3139850
32	No	Show	32	H	4.7668960	0.4778220	2.2163360
33	No	Show	33	H	5.0475080	-1.3697030	-1.6828460
34	No	Show	34	H	6.0974980	-0.8292710	0.5339390
35	No	Show	35	N	2.5062790	0.2660450	-0.2433910

Compound 23

Row	Highlight	Display	Tag	Symbol	X	Y	Z
1	No	Show	1	C	-0.9360360	1.9615260	-0.7445880
2	No	Show	2	C	-1.8361460	2.1481340	0.3472960
3	No	Show	3	H	-1.0245100	2.3995550	-1.7303900
4	No	Show	4	C	-1.4050530	1.3864810	1.4808830
5	No	Show	5	H	-2.7483410	2.7300680	0.3036820
6	No	Show	6	H	-1.9169410	1.3101700	2.4313060
7	No	Show	7	C	0.1068340	1.1036860	-0.2577750
8	No	Show	8	H	-2.8055160	-0.1544310	-2.7044490
9	No	Show	9	C	-2.7262150	-0.6155680	-1.7279210
10	No	Show	10	C	-3.5884570	-0.3818130	-0.6162280
11	No	Show	11	C	-1.7285550	-1.5525680	-1.3170360
12	No	Show	12	C	-3.1142250	-1.1569840	0.4836800
13	No	Show	13	H	-4.4297150	0.2997790	-0.5970490
14	No	Show	14	C	-1.9627410	-1.8824850	0.0483650
15	No	Show	15	H	-0.9175690	-1.9229700	-1.9325360
16	No	Show	16	H	-1.3667590	-2.5483860	0.6606640
17	No	Show	17	Fe	-1.6706040	0.1497920	-0.1434620
18	No	Show	18	H	-3.5365250	-1.1735690	1.4806810
19	No	Show	19	C	-0.1947860	0.7407150	1.1013220
20	No	Show	20	C	0.8335210	-0.1945050	1.5605540
21	No	Show	21	O	1.0608140	-0.7701760	2.5887370
22	No	Show	22	N	1.7059720	-0.3580080	0.3628460
23	No	Show	23	C	2.8665570	-1.2546160	0.4094550
24	No	Show	24	C	1.2531880	0.3567450	-0.6549350
25	No	Show	25	H	1.7427480	0.3489850	-1.6227860
26	No	Show	26	C	3.8865900	-1.0151040	-0.6917180
27	No	Show	27	H	4.7125710	-1.7065900	-0.5101200
28	No	Show	28	H	3.4719100	-1.2486430	-1.6773850
29	No	Show	29	H	3.3140960	-1.1202960	1.3971680
30	No	Show	30	S	4.4963220	0.7117080	-0.6389410
31	No	Show	31	H	5.3390360	0.5969180	-1.6812170
32	No	Show	32	H	2.5048860	-2.2869090	0.3575490

Compound 24

Row	Highlight	Display	Tag	Symbol	X	Y	Z
1	No	Show	1	C	1.4138540	-0.7704130	-1.9211650

2	No	Show	2	C	2.4342890	-1.3791810	-1.1299000
3	No	Show	3	H	1.5472130	-0.2942440	-2.8839440
4	No	Show	4	C	1.8827120	-1.8743360	0.0953390
5	No	Show	5	H	3.4855120	-1.4022980	-1.3888900
6	No	Show	6	H	2.4322680	-2.3474570	0.8986780
7	No	Show	7	C	0.1887510	-0.9437160	-1.1921510
8	No	Show	8	H	2.1332480	2.5348100	-1.4252610
9	No	Show	9	C	2.0054590	2.0931200	-0.4448760
10	No	Show	10	C	3.0173000	1.4400860	0.3198540
11	No	Show	11	C	0.7896570	2.0430750	0.3038220
12	No	Show	12	C	2.4238060	0.9686090	1.5285230
13	No	Show	13	H	4.0453040	1.2868600	0.0157190
14	No	Show	14	C	1.0451140	1.3447480	1.5181120
15	No	Show	15	H	-0.1712110	2.4260640	-0.0181510
16	No	Show	16	H	0.3202570	1.1178060	2.2907070
17	No	Show	17	Fe	1.4950570	0.1384730	-0.0941580
18	No	Show	18	H	2.9239800	0.4047360	2.3060690
19	No	Show	19	C	0.4855610	-1.6044790	0.0516220
20	No	Show	20	C	-0.7560110	-1.7078620	0.8184710
21	No	Show	21	O	-1.0629340	-2.1671390	1.8838240
22	No	Show	22	N	-1.7600850	-1.0415600	-0.0643110
23	No	Show	23	C	-3.1591700	-0.9346160	0.3653550
24	No	Show	24	C	-1.1895030	-0.5862200	-1.1680650
25	No	Show	25	H	-1.7506510	-0.0392550	-1.9184770
26	No	Show	26	C	-3.9778570	0.1019950	-0.3841760
27	No	Show	27	H	-4.0400890	-0.1311990	-1.4514570
28	No	Show	28	H	-4.9906400	0.0569560	0.0223670
29	No	Show	29	H	-3.6182980	-1.9206960	0.2400600
30	No	Show	30	S	-3.2901320	1.7800660	-0.1249880
31	No	Show	31	H	-4.2046810	2.4349790	-0.8622550
32	No	Show	32	H	-3.1412980	-0.7252560	1.4377520

Compound 25

Row	Highlight		Display	Tag	Symbol X	Y	Z
1	No	Show	1	H	-3.6490580	-2.4141940	-0.3482990
2	No	Show	2	C	-2.6922170	-1.9441900	-0.5344450
3	No	Show	3	C	-2.1804380	-1.5048200	-1.7852670
4	No	Show	4	C	-1.7393320	-1.6202590	0.4846870
5	No	Show	5	C	-0.9077030	-0.9015040	-1.5619650
6	No	Show	6	H	-2.6901460	-1.5637500	-2.7391250
7	No	Show	7	C	-0.6157290	-0.9641940	-0.1579910
8	No	Show	8	Fe	-2.3281430	0.0677940	-0.4699080
9	No	Show	9	C	-2.3853550	1.9846330	-1.1698840
10	No	Show	10	C	-2.0277540	1.9722920	0.2124340
11	No	Show	11	C	-3.6651900	1.3623300	-1.3012800
12	No	Show	12	H	-1.7719920	2.3577550	-1.9808530
13	No	Show	13	C	-3.0872620	1.3453160	0.9354070

14	No	Show	14	H	-1.0893770	2.3191040	0.6275810
15	No	Show	15	C	-4.0991920	0.9658080	0.0013810
16	No	Show	16	H	-4.1931030	1.1822900	-2.2297950
17	No	Show	17	H	-3.0983530	1.1428500	1.9991500
18	No	Show	18	H	-5.0115990	0.4315260	0.2355300
19	No	Show	19	H	-0.3052340	-0.4185310	-2.3220620
20	No	Show	20	C	-1.9591970	-1.8729770	1.9216990
21	No	Show	21	O	-2.9491860	-2.4532360	2.3430130
22	No	Show	22	H	-1.1708340	-1.5053430	2.5995690
23	No	Show	23	C	0.5940790	-0.4462910	0.5311170
24	No	Show	24	O	0.6485190	-0.2668730	1.7481850
25	No	Show	25	N	1.6587550	-0.1776920	-0.2924230
26	No	Show	26	H	1.6761970	-0.5910050	-1.2178210
27	No	Show	27	C	2.9459740	0.1733620	0.2556630
28	No	Show	28	C	3.3123480	1.6635210	0.0840250
29	No	Show	29	H	3.3545000	1.9182760	-0.9792160
30	No	Show	30	H	4.3005430	1.8442000	0.5131610
31	No	Show	31	S	2.1068570	2.8288110	0.7925950
32	No	Show	32	H	2.1324300	2.3389350	2.0471200
33	No	Show	33	H	2.9371450	-0.0636890	1.3238540
34	No	Show	34	C	4.0118110	-0.6723140	-0.4327480
35	No	Show	35	O	3.8394820	-1.2653020	-1.4793020
36	No	Show	36	O	5.1698570	-0.6459180	0.2396730
37	No	Show	37	C	6.2534100	-1.3808840	-0.3611740
38	No	Show	38	H	6.4796560	-0.9812930	-1.3514990
39	No	Show	39	H	7.1000090	-1.2484910	0.3101600
40	No	Show	40	H	5.9910180	-2.4367530	-0.4469120

Compound 26

Row	Highlight		Display Tag	Symbol X	Y	Z	
1	No	Show	1	H	-3.8460820	1.5895080	1.4878940
2	No	Show	2	C	-2.8629490	1.1527510	1.3705940
3	No	Show	3	C	-2.2602630	0.1600900	2.1899060
4	No	Show	4	C	-1.9610940	1.4671070	0.3027210
5	No	Show	5	C	-0.9807220	-0.1554470	1.6450270
6	No	Show	6	H	-2.7132830	-0.3172330	3.0501510
7	No	Show	7	C	-0.7783570	0.6450930	0.4720030
8	No	Show	8	Fe	-2.4303710	-0.5022630	0.2505080
9	No	Show	9	C	-2.3287550	-2.4847850	-0.2269030
10	No	Show	10	C	-2.1408930	-1.6814750	-1.3929140
11	No	Show	11	C	-3.6073840	-2.1657400	0.3264170
12	No	Show	12	H	-1.6102940	-3.1796440	0.1904950
13	No	Show	13	C	-3.3031310	-0.8698860	-1.5617210
14	No	Show	14	H	-1.2521560	-1.6442670	-2.0104990
15	No	Show	15	C	-4.2093950	-1.1662960	-0.4990830
16	No	Show	16	H	-4.0281010	-2.5780280	1.2353340
17	No	Show	17	H	-3.4471230	-0.1185230	-2.3279900

18	No	Show	18	H	-5.1636820	-0.6846980	-0.3258580
19	No	Show	19	H	-0.3128240	-0.9203890	2.0240350
20	No	Show	20	C	-2.2670740	2.4166980	-0.7837030
21	No	Show	21	O	-3.2997460	3.0694460	-0.8176520
22	No	Show	22	H	-1.5010060	2.5053590	-1.5728440
23	No	Show	23	C	0.4019150	0.6264550	-0.4317750
24	No	Show	24	O	0.3610850	1.0027430	-1.6065560
25	No	Show	25	N	1.5549490	0.1648200	0.1300960
26	No	Show	26	H	1.5983330	-0.0834800	1.1098240
27	No	Show	27	C	2.7681250	0.0440090	-0.6375500
28	No	Show	28	H	2.5007180	-0.2879910	-1.6484440
29	No	Show	29	C	3.5235510	1.3768400	-0.8051260
30	No	Show	30	H	2.8334340	2.0901910	-1.2603440
31	No	Show	31	H	4.3685330	1.2477830	-1.4840250
32	No	Show	32	S	4.0813440	2.1512810	0.7478750
33	No	Show	33	H	5.1565180	1.3627810	0.9473760
34	No	Show	34	C	3.6322090	-1.0347070	-0.0031730
35	No	Show	35	O	3.3619600	-1.6225010	1.0236790
36	No	Show	36	O	4.7439600	-1.2479250	-0.7248660
37	No	Show	37	C	5.6287960	-2.2623130	-0.2143990
38	No	Show	38	H	6.4647900	-2.2948300	-0.9109920
39	No	Show	39	H	5.9718010	-1.9998210	0.7880910
40	No	Show	40	H	5.1183740	-3.2265830	-0.1808310

Compound 27

Row	Highlight	Display	Tag	Symbol X	Y	Z	
1	No	Show	1	C	2.0738190	-1.7685670	-1.1234390
2	No	Show	2	C	2.9569740	-1.9224310	-0.0134610
3	No	Show	3	H	2.3174090	-1.9338000	-2.1648640
4	No	Show	4	C	2.2851730	-1.5864730	1.2059770
5	No	Show	5	H	4.0040570	-2.1882510	-0.0888090
6	No	Show	6	H	2.7248640	-1.5743080	2.1947440
7	No	Show	7	C	0.8044680	-1.3844280	-0.5711860
8	No	Show	8	H	3.1357730	1.2028020	-2.4735490
9	No	Show	9	C	2.9524710	1.3819840	-1.4213930
10	No	Show	10	C	3.8754200	1.1491150	-0.3587800
11	No	Show	11	C	1.7384190	1.8737020	-0.8490560
12	No	Show	12	C	3.2265620	1.4755630	0.8695820
13	No	Show	13	H	4.8766200	0.7491800	-0.4613290
14	No	Show	14	C	1.9056080	1.9263310	0.5632260
15	No	Show	15	H	0.8255350	2.1136630	-1.3795850
16	No	Show	16	H	1.1532370	2.2253850	1.2823310
17	No	Show	17	Fe	2.2041480	-0.0018310	-0.1073680
18	No	Show	18	H	3.6518400	1.3748510	1.8604360
19	No	Show	19	C	0.9467530	-1.2527530	0.8563350
20	No	Show	20	C	-0.3270680	-0.7792240	1.3999650
21	No	Show	21	O	-0.7381840	-0.5185350	2.4975810
22	No	Show	22	N	-1.1792300	-0.6382110	0.1878930

23	No	Show	23	C	-2.5333720	-0.1106030	0.2726790
24	No	Show	24	C	-0.4959530	-0.9300440	-0.9120570
25	No	Show	25	H	-0.9400420	-0.8368060	-1.8973600
26	No	Show	26	C	-2.5236780	1.3864300	-0.0740010
27	No	Show	27	O	-1.6362810	1.9196000	-0.7076870
28	No	Show	28	O	-3.6221970	1.9704800	0.3814970
29	No	Show	29	C	-3.7752810	3.3808060	0.0811160
30	No	Show	30	H	-4.7320980	3.6610500	0.5147670
31	No	Show	31	H	-2.9620680	3.9454720	0.5380380
32	No	Show	32	H	-3.7761010	3.5331270	-0.9986780
33	No	Show	33	C	-3.5277880	-0.8420160	-0.6394270
34	No	Show	34	H	-4.5281300	-0.5173910	-0.3483980
35	No	Show	35	H	-3.3711400	-0.5719140	-1.6881830
36	No	Show	36	H	-2.8344050	-0.2320280	1.3168520
37	No	Show	37	S	-3.3565440	-2.6508540	-0.4150420
38	No	Show	38	H	-4.4157500	-2.9790090	-1.1770070

Compound 28

Row	Highlight		Display	Tag	Symbol X	Y	Z
1	No	Show	1	C	2.0291950	-1.8022950	-1.3470510
2	No	Show	2	C	2.6216050	-2.2991660	-0.1479830
3	No	Show	3	H	2.4424800	-1.8743160	-2.3447450
4	No	Show	4	C	1.7926110	-1.9933690	0.9790000
5	No	Show	5	H	3.5903440	-2.7796270	-0.0906090
6	No	Show	6	H	2.0140670	-2.2180240	2.0142260
7	No	Show	7	C	0.7716130	-1.2261410	-0.9589080
8	No	Show	8	H	4.0424240	0.9215220	-1.8839590
9	No	Show	9	C	3.6227110	1.0320770	-0.8918490
10	No	Show	10	C	4.1601310	0.4870290	0.3121340
11	No	Show	11	C	2.4230120	1.7312580	-0.5566220
12	No	Show	12	C	3.2834410	0.8314040	1.3840310
13	No	Show	13	H	5.0519150	-0.1222990	0.3918140
14	No	Show	14	C	2.2079580	1.6026990	0.8446550
15	No	Show	15	H	1.7664750	2.2387780	-1.2525210
16	No	Show	16	H	1.3616230	1.9976560	1.3928680
17	No	Show	17	Fe	2.2899620	-0.2656480	-0.0256670
18	No	Show	18	H	3.3985060	0.5361970	2.4195840
19	No	Show	19	C	0.6428600	-1.3268770	0.4710100
20	No	Show	20	C	-0.6160260	-0.6920760	0.8642220
21	No	Show	21	O	-1.1970930	-0.5397970	1.9026170
22	No	Show	22	N	-1.1568100	-0.1822360	-0.4323160
23	No	Show	23	C	-2.4749420	0.4426970	-0.5474300
24	No	Show	24	C	-0.3445900	-0.4871030	-1.4326170
25	No	Show	25	H	-0.5809880	-0.2027260	-2.4542500
26	No	Show	26	C	-3.5292740	-0.6413370	-0.2379070
27	No	Show	27	O	-3.2789260	-1.8233370	-0.2900020
28	No	Show	28	O	-4.7118640	-0.0945760	0.0161640
29	No	Show	29	C	-5.7977560	-1.0258690	0.2591130

30	No	Show	30	H	-6.6710430	-0.4032960	0.4384180
31	No	Show	31	H	-5.9432730	-1.6606550	-0.6154380
32	No	Show	32	H	-5.5683400	-1.6385370	1.1312050
33	No	Show	33	C	-2.6161330	1.7226640	0.2762910
34	No	Show	34	H	-3.6023710	2.1373610	0.0685690
35	No	Show	35	H	-2.5322530	1.5131090	1.3425620
36	No	Show	36	H	-2.6046200	0.7000270	-1.6049610
37	No	Show	37	S	-1.3282840	2.9096710	-0.2598920
38	No	Show	38	H	-1.9091730	4.0107170	0.2483090

Compound 29

Row	Highlight		Display	Tag	Symbol X	Y	Z
1	No	Show	1	C	1.7784950	-1.9650940	-1.1635270
2	No	Show	2	C	2.6047460	-2.3169280	-0.0546630
3	No	Show	3	H	1.9862940	-2.1629950	-2.2070760
4	No	Show	4	C	2.0124260	-1.8654890	1.1687320
5	No	Show	5	H	3.5722980	-2.7969370	-0.1324690
6	No	Show	6	H	2.4390210	-1.9619600	2.1586370
7	No	Show	7	C	0.6158560	-1.3312220	-0.6076130
8	No	Show	8	H	3.4608980	0.7314110	-2.4604050
9	No	Show	9	C	3.3114100	0.9305050	-1.4065070
10	No	Show	10	C	4.1539410	0.4911730	-0.3425380
11	No	Show	11	C	2.2260690	1.6616930	-0.8314170
12	No	Show	12	C	3.5795880	0.9310720	0.8875140
13	No	Show	13	H	5.0477220	-0.1116260	-0.4462870
14	No	Show	14	C	2.3873440	1.6571580	0.5825300
15	No	Show	15	H	1.3978570	2.1097780	-1.3666430
16	No	Show	16	H	1.7093960	2.0992870	1.3020580
17	No	Show	17	Fe	2.2745650	-0.2806190	-0.1187760
18	No	Show	18	H	3.9657580	0.7288760	1.8787980
19	No	Show	19	C	0.7753670	-1.2539220	0.8213610
20	No	Show	20	C	-0.3848670	-0.5523290	1.3723730
21	No	Show	21	O	-0.7616360	-0.2740170	2.4771890
22	No	Show	22	N	-1.1691470	-0.1895230	0.1573810
23	No	Show	23	S	-1.4336490	2.9465180	0.1030570
24	No	Show	24	C	-2.6434070	1.7234810	-0.5191320
25	No	Show	25	H	-2.5106150	1.5605210	-1.5926810
26	No	Show	26	H	-3.6576890	2.0884060	-0.3471810
27	No	Show	27	C	-2.4931170	0.4081930	0.2542050
28	No	Show	28	C	-0.5749540	-0.6331160	-0.9442900
29	No	Show	29	H	-1.0018210	-0.4679520	-1.9270460
30	No	Show	30	H	-2.6650890	0.5889650	1.3190600
31	No	Show	31	C	-3.5239400	-0.6111380	-0.2666180
32	No	Show	32	O	-3.2429880	-1.4888520	-1.0555700
33	No	Show	33	O	-4.7225670	-0.3582610	0.2353590
34	No	Show	34	C	-5.7996180	-1.2224230	-0.2121450
35	No	Show	35	H	-6.6867500	-0.8568300	0.2990560

36	No	Show	36	H	-5.9113410	-1.1457440	-1.2941190
37	No	Show	37	H	-5.5839970	-2.2532980	0.0696120
38	No	Show	38	H	-1.9127120	3.9879320	-0.6011390

Compound 30

Row	Highlight	Display	Tag	Symbol	X	Y	Z
1	No	Show	1	C	2.2616620	-1.9132250	-0.8437120
2	No	Show	2	C	2.9247690	-1.9278350	0.4195060
3	No	Show	3	H	2.6881120	-2.2010610	-1.7958250
4	No	Show	4	C	2.0482180	-1.4498990	1.4462200
5	No	Show	5	H	3.9632870	-2.1956450	0.5690710
6	No	Show	6	H	2.3009160	-1.3158520	2.4899520
7	No	Show	7	C	0.9190740	-1.4726260	-0.5820680
8	No	Show	8	H	3.6421250	0.8819870	-2.2975160
9	No	Show	9	C	3.2517930	1.1853220	-1.3340200
10	No	Show	10	C	3.9361220	1.0938720	-0.0856290
11	No	Show	11	C	1.9550630	1.7313360	-1.0811100
12	No	Show	12	C	3.0564360	1.5622780	0.9361570
13	No	Show	13	H	4.9314190	0.6949870	0.0665340
14	No	Show	14	C	1.8305690	1.9576240	0.3180000
15	No	Show	15	H	1.1781100	1.9057930	-1.8149940
16	No	Show	16	H	0.9436000	2.3254780	0.8175960
17	No	Show	17	Fe	2.2328630	-0.0359990	-0.0398640
18	No	Show	18	H	3.2714260	1.5870180	1.9972420
19	No	Show	19	C	0.8010540	-1.1693010	0.8211610
20	No	Show	20	C	-0.5335000	-0.6186950	1.0607360
21	No	Show	21	O	-1.1267050	-0.2241670	2.0269930
22	No	Show	22	N	-1.1440010	-0.6115520	-0.2999740
23	No	Show	23	S	-3.4010220	-2.6362740	-0.3505630
24	No	Show	24	C	-3.5731480	-0.8822410	0.1489310
25	No	Show	25	H	-3.4960700	-0.7765940	1.2314180
26	No	Show	26	H	-4.5364650	-0.4937350	-0.1816280
27	No	Show	27	C	-2.4802240	-0.0756620	-0.5562090
28	No	Show	28	C	-0.2807480	-1.0458230	-1.2073500
29	No	Show	29	H	-0.5381980	-1.0687910	-2.2624660
30	No	Show	30	H	-2.6424840	-0.1547100	-1.6374870
31	No	Show	31	C	-2.4680620	1.4296350	-0.2367920
32	No	Show	32	O	-1.4508010	2.0898180	-0.2533330
33	No	Show	33	O	-3.6932810	1.8854910	-0.0218200
34	No	Show	34	C	-3.8108010	3.3094920	0.2248020
35	No	Show	35	H	-4.8755800	3.4886230	0.3529290
36	No	Show	36	H	-3.2604750	3.5722510	1.1286090
37	No	Show	37	H	-3.4210030	3.8670910	-0.6273430
38	No	Show	38	H	-4.6421700	-3.0142760	0.0027400

TS(24-23)

Row	Highlight	Display	Tag	Symbol	X	Y	Z
1	No	Show	6	C	-1.8825970	-1.9513850	0.3272150

2	No	Show	9	C	-2.2972320	-1.6792090	-1.0234320
3	No	Show	14	H	-2.4373870	-2.5138500	1.0589530
4	No	Show	4	C	-1.2607320	-0.9750020	-1.7311650
5	No	Show	19	H	-3.2521090	-1.9547560	-1.4414580
6	No	Show	11	H	-1.2889410	-0.6701680	-2.7642860
7	No	Show	3	C	-0.5242320	-1.4751250	0.4337410
8	No	Show	23	H	-3.6720950	0.4437290	2.0451920
9	No	Show	15	C	-3.0665230	0.9010420	1.2799780
10	No	Show	7	C	-3.4655480	1.1459620	-0.0782010
11	No	Show	22	C	-1.7538050	1.4569620	1.4590500
12	No	Show	8	C	-2.3928350	1.8344990	-0.7405190
13	No	Show	16	H	-4.4202480	0.8964040	-0.5120290
14	No	Show	17	C	-1.3340850	2.0277310	0.2141430
15	No	Show	28	H	-1.1977340	1.4745350	2.3829730
16	No	Show	24	H	-0.4091840	2.5521780	0.0326070
17	No	Show	1	Fe	-1.7654710	0.0298400	-0.0381770
18	No	Show	18	H	-2.4026520	2.1958450	-1.7559060
19	No	Show	2	C	-0.1655840	-0.8325680	-0.8193820
20	No	Show	5	C	1.2131080	-0.3937500	-0.7572990
21	No	Show	13	O	1.9832470	0.1523910	-1.5160630
22	No	Show	12	N	1.6176440	-0.7965990	0.6714690
23	No	Show	21	C	2.9245420	-0.4776950	1.3150270
24	No	Show	10	C	0.6048530	-1.3879040	1.2891520
25	No	Show	20	H	0.6903700	-1.7503530	2.3068240
26	No	Show	25	C	4.1260060	-0.5351690	0.3580410
27	No	Show	29	H	5.0069030	-0.8585100	0.9171200
28	No	Show	30	H	3.9635960	-1.2402940	-0.4571110
29	No	Show	26	H	2.8499110	0.5232560	1.7536570
30	No	Show	31	S	4.5178690	1.1455050	-0.4252510
31	No	Show	32	H	5.5970360	1.4186160	0.3819270
32	No	Show	27	H	3.0473810	-1.2138230	2.1182810

TS(28-27)

Row	Highlight	Display	Tag	Symbol	X	Y	Z
1	No	Show	1	C	-2.2685720	-1.3928410	-1.5646280
2	No	Show	2	C	-3.1241510	-0.2899500	-1.8624530
3	No	Show	3	H	-2.5330970	-2.4413750	-1.6088450
4	No	Show	4	C	-2.4286350	0.9461000	-1.6615760
5	No	Show	5	H	-4.1683620	-0.3740130	-2.1367070
6	No	Show	6	H	-2.8442160	1.9390340	-1.7740600
7	No	Show	7	C	-0.9944530	-0.8244640	-1.2242330
8	No	Show	8	H	-3.4611360	-2.3366230	1.5267020
9	No	Show	9	C	-3.2301040	-1.2801890	1.5830750
10	No	Show	10	C	-4.0971760	-0.2104270	1.2118950
11	No	Show	11	C	-1.9971660	-0.7088670	2.0257530
12	No	Show	12	C	-3.3957020	1.0168080	1.4060450

13	No	Show	13	H	-5.0975370	-0.3131410	0.8100780
14	No	Show	14	C	-2.0953650	0.7071730	1.9103530
15	No	Show	15	H	-1.1295970	-1.2628990	2.3636710
16	No	Show	16	H	-1.3176480	1.4257180	2.1400290
17	No	Show	17	Fe	-2.4015510	-0.1706460	0.0685740
18	No	Show	18	H	-3.7719640	2.0080430	1.1860300
19	No	Show	19	C	-1.1042790	0.6059800	-1.2679510
20	No	Show	20	C	0.1646950	1.1784510	-0.8214170
21	No	Show	21	O	0.5594560	2.3013480	-0.6780170
22	No	Show	22	N	1.0018970	-0.0310030	-0.5131890
23	No	Show	23	C	2.3447000	0.0563840	0.0907410
24	No	Show	24	C	0.2855100	-1.1373200	-0.6937450
25	No	Show	25	H	0.6886620	-2.1170820	-0.4702270
26	No	Show	26	C	3.1055620	-1.2657260	-0.0708620
27	No	Show	27	O	2.8501930	-2.1331190	-0.8801590
28	No	Show	28	O	4.1173740	-1.2877780	0.7901910
29	No	Show	29	C	5.0039850	-2.4329150	0.7050490
30	No	Show	30	H	5.7528640	-2.2691220	1.4761050
31	No	Show	31	H	4.4449570	-3.3498810	0.8935930
32	No	Show	32	H	5.4634890	-2.4741260	-0.2830010
33	No	Show	33	C	3.2126020	1.2005730	-0.5145020
34	No	Show	34	H	4.2236960	0.8277810	-0.6770020
35	No	Show	35	H	2.8281570	1.4891090	-1.4925470
36	No	Show	36	H	2.2232160	0.2440760	1.1614390
37	No	Show	37	S	3.3220120	2.7024890	0.5122350
38	No	Show	38	H	4.4684680	2.3622680	1.1329800

TS(30-29)

Row	Highlight		Display	Tag	Symbol X	Y	Z
1	No	Show	1	C	-2.3757060	-0.8892970	-1.8546140
2	No	Show	2	C	-3.2314860	0.2506360	-1.7800510
3	No	Show	3	H	-2.6640020	-1.8906700	-2.1471300
4	No	Show	4	C	-2.4973560	1.3932400	-1.3252730
5	No	Show	5	H	-4.2963550	0.2379690	-1.9764090
6	No	Show	6	H	-2.9012710	2.3801450	-1.1404930
7	No	Show	7	C	-1.0661280	-0.4257840	-1.4884630
8	No	Show	8	H	-3.2796780	-2.6447480	0.9278140
9	No	Show	9	C	-3.0392640	-1.6417080	1.2575940
10	No	Show	10	C	-3.9306150	-0.5282640	1.2790080
11	No	Show	11	C	-1.7659400	-1.1934040	1.7276700
12	No	Show	12	C	-3.2061360	0.6102570	1.7417370
13	No	Show	13	H	-4.9641440	-0.5345540	0.9556340
14	No	Show	14	C	-1.8675720	0.1958000	2.0222200
15	No	Show	15	H	-0.8695910	-1.7976830	1.8042590
16	No	Show	16	H	-1.0698980	0.8374640	2.3758750
17	No	Show	17	Fe	-2.3459740	-0.1427850	0.0441970
18	No	Show	18	H	-3.5949830	1.6159500	1.8416110
19	No	Show	19	C	-1.1509080	0.9668910	-1.1494700

20	No	Show	20	C	0.1683290	1.4088410	-0.6982510
21	No	Show	21	O	0.6234450	2.4694200	-0.3735890
22	No	Show	22	N	0.9840510	0.1473430	-0.7243830
23	No	Show	23	S	3.5691130	2.5529780	0.6020940
24	No	Show	24	C	2.7395800	0.9617270	0.9110540
25	No	Show	25	H	1.8233160	1.1705390	1.4625280
26	No	Show	26	H	3.3681830	0.3696560	1.5771620
27	No	Show	27	C	2.4151450	0.1278640	-0.3685900
28	No	Show	28	C	0.2446590	-0.8667480	-1.1622280
29	No	Show	29	H	0.6505550	-1.8674270	-1.2482400
30	No	Show	30	H	2.9683320	0.5496050	-1.2116490
31	No	Show	31	C	2.9108290	-1.2992570	-0.1271630
32	No	Show	32	O	2.2218520	-2.2263200	0.2513710
33	No	Show	33	O	4.2223420	-1.3434860	-0.3250180
34	No	Show	34	C	4.8757180	-2.6028140	-0.0227210
35	No	Show	35	H	5.9300230	-2.4300120	-0.2243150
36	No	Show	36	H	4.7153950	-2.8634700	1.0239480
37	No	Show	37	H	4.4804560	-3.3884380	-0.6672040
38	No	Show	38	H	4.8247940	2.0842330	0.7377910

Pyridine

Row	Highlight	Display	Tag	Symbol	X	Y	Z
1	No	Show	1	C	-1.1413930	-0.7217640	-0.0002170
2	No	Show	2	C	-1.1978510	0.6729370	-0.0001220
3	No	Show	3	C	0.0002690	1.3856700	0.0000990
4	No	Show	4	C	1.1981140	0.6724870	0.0002150
5	No	Show	5	C	1.1411130	-0.7221910	0.0001220
6	No	Show	6	N	-0.0002760	-1.4199540	-0.0000950
7	No	Show	7	H	0.0004500	2.4727800	0.0001660
8	No	Show	8	H	-2.0590550	-1.3088300	-0.0003740
9	No	Show	9	H	-2.1577230	1.1816780	-0.0002420
10	No	Show	10	H	2.1582010	1.1808270	0.0003520
11	No	Show	11	H	2.0585430	-1.3096150	0.0001820

Protopyridinium ion

Row	Highlight	Display	Tag	Symbol	X	Y	Z
1	No	Show	1	C	-0.6664220	1.1877310	-0.0002080
2	No	Show	2	C	0.7163500	1.2099510	-0.0001200
3	No	Show	3	C	1.4147020	0.0000040	0.0000940
4	No	Show	4	C	0.7163560	-1.2099470	0.0002140
5	No	Show	5	C	-0.6664160	-1.1877340	0.0001200
6	No	Show	6	N	-1.3082650	-0.0000030	-0.0000860
7	No	Show	7	H	2.5007760	0.0000070	0.0001650
8	No	Show	8	H	-1.2840570	2.0789110	-0.0003720
9	No	Show	9	H	1.2323160	2.1638470	-0.0002190
10	No	Show	10	H	1.2323270	-2.1638410	0.0003810

11	No	Show	11	H	-1.2840460	-2.0789170	0.0002020
12	No	Show	12	H	-2.3268840	-0.0000060	-0.0001530

Imidazole

Row	Highlight	Display	Tag	Symbol	X	Y	Z
1	No	Show	1	N	-0.7785310	0.7845930	-0.0009070
2	No	Show	2	C	-0.9723990	-0.5669610	0.0002180
3	No	Show	3	C	0.5823890	1.0022250	0.0004350
4	No	Show	4	H	-1.9592410	-1.0124050	-0.0002910
5	No	Show	5	C	1.1434730	-0.2528370	0.0001530
6	No	Show	6	H	1.0099630	1.9947480	-0.0010850
7	No	Show	7	H	2.1952590	-0.5092750	-0.0003430
8	No	Show	8	N	0.1686320	-1.2219710	-0.0000100
9	No	Show	9	H	-1.4974650	1.4940140	0.0033060

Protoimidazolium ion

Row	Highlight	Display	Tag	Symbol	X	Y	Z
1	No	Show	1	N	-1.0750780	-0.3419080	-0.0001100
2	No	Show	2	C	-0.0062530	-1.1403800	0.0000230
3	No	Show	3	C	-0.6763520	0.9795030	0.0000540
4	No	Show	4	H	-0.0121100	-2.2209830	-0.0000730
5	No	Show	5	C	0.6870380	0.9722670	-0.0000140
6	No	Show	6	H	-1.3797470	1.7991760	-0.0000820
7	No	Show	7	H	1.3994340	1.7841570	-0.0000280
8	No	Show	8	N	1.0712530	-0.3535060	0.0000380
9	No	Show	9	H	-2.0362340	-0.6704980	0.0003840
10	No	Show	10	H	2.0288350	-0.6922870	-0.0000640

H_2O

Row	Highlight	Display	Tag	Symbol	X	Y	Z
1	No	Show	1	O	0.0000000	0.0000000	0.1172430
2	No	Show	2	H	0.0000000	0.7684870	-0.4689710
3	No	Show	3	H	0.0000000	-0.7684870	-0.4689710
